# Automated Training of Deep Convolutional Neural Networks for Cell Segmentation

**DOI:** 10.1038/s41598-017-07599-6

**Published:** 2017-08-10

**Authors:** Sajith Kecheril Sadanandan, Petter Ranefall, Sylvie Le Guyader, Carolina Wählby

**Affiliations:** 1 Department of Information Technology, Uppsala University, Sweden and SciLifeLab, Uppsala, Sweden; 2 Center for Biosciences, Department of Biosciences and Nutrition, Novum, Karolinska Institutet, Huddinge, Sweden

## Abstract

Deep Convolutional Neural Networks (DCNN) have recently emerged as superior for many image segmentation tasks. The DCNN performance is however heavily dependent on the availability of large amounts of problem-specific training samples. Here we show that DCNNs trained on ground truth created automatically using fluorescently labeled cells, perform similar to manual annotations.

## Introduction

Experiments where live cell samples are imaged over prolonged periods of time have the potential to help us understand how cells behave and respond to changes in the local environment. Modern imaging experiments, with ever increasing scale, rule out manual/visual image processing and analysis. A central problem in many automated analysis approaches, especially those following individual cells over time, is image segmentation. Bright-field imaging is currently the least invasive microscopy imaging approach allowing long time-lapse studies, it is available on all microscopes, and is the cheapest microscopy method to implement. However, automated segmentation of unstained cells imaged by bright-field microscopy is typically very challenging, and many of the common approaches, as reviewed by Meijering^1^, fail.

Recently, it has been shown that methods based on Deep Convolutional Neural Networks (DCNNs)^2–4^ solve segmentation problems very difficult to solve using traditional image processing methods. Traditional image segmentation approaches often require experiment-specific parameter tuning, while DCNNs instead require training data. Large amounts of high-quality annotated samples, or ground truth is typically required for DCNN training. The ground truth represents the extent to which an object is present in an image in reality. In an image the extent of a particular object is specified by the boundary of the object, usually created by a domain expert. Visual/manual annotation is tedious, cumbersome and expensive. The annotated samples for one dataset may not be useful for another dataset, and new ground truth generation may be needed for every new dataset, limiting the usability of DCNNs. Manually created ground truths have been used to segment both bacterial and mammalian cells^5^. Automated training has previously been suggested for classification of cells from imaging flow cytometry data where each image contains a single cell in liquid solution^6^.

**Figure 1:**
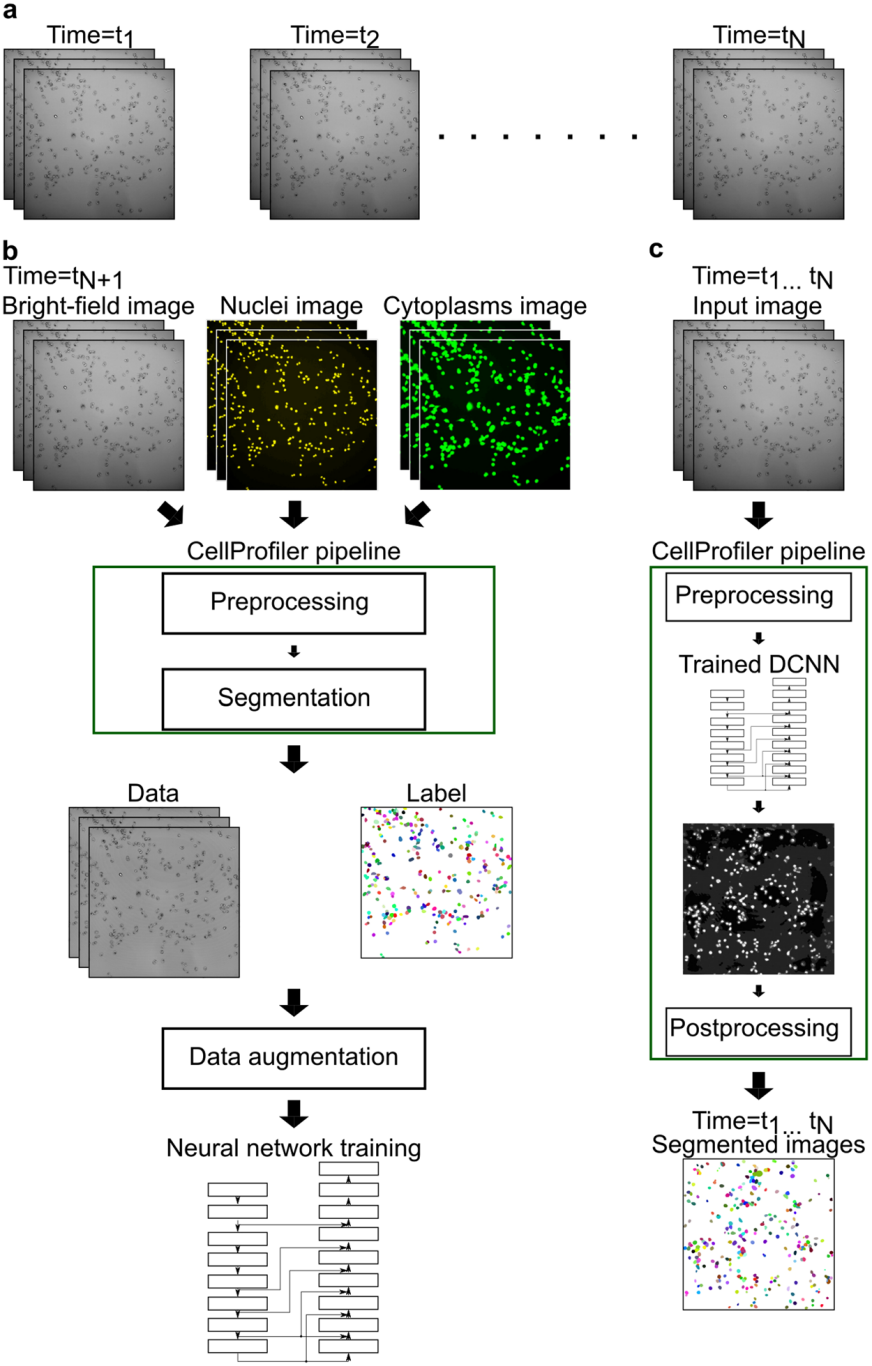
Data, training and testing pipelines for cell segmentation. (**a**) A z-stack of bright-field time-lapse microscopy images captured without any staining (time = *t*_1_ to *t*_*N*_). (**b**) A z-stack of bright-field images along with fluorescent images of nuclear and cytoplasmic stains captured after staining (time = *t*_*N*+1_). The green box represents the CellProfiler pipeline automatically generating illumination corrected images (data) and the segmented images (labels) for DCNN training. The data and the label are subjected to data augmentation to create the final dataset for training the DCNN. See Supplementary Fig. 2 for the detailed DCNN architecture. (**c**) The complete cell segmentation pipeline in CellProfiler (green box) that receives z-stack bright-field input images and outputs segmented cell images.

We propose an automated approach for creation of high-quality experiment-specific ground truth for segmentation of bright-field images of cultured cells based on end-point fluorescent staining. We capture bright-field images during the entire period of the experiment (Fig. 1a). At the end of the experiment, we stain the cells with fluorescent markers for nuclei and cytoplasmic regions. After staining, we capture fluorescent images along with bright-field data (Fig. 1b, Supplementary Fig. 1). We apply standard automated methods available in CellProfiler^7^ to segment the cells based on the information from the fluorescent channels. Finally, we use these automatically created segmentation results as labels along with the corresponding bright-field image data to train a DCNN (Fig. 1b). To handle slight variations in focal depth, inspired by Liimatainen *et al*.^8^, we collected bright-field data in a z-stack at three consecutive focus levels both for training and for the time-lapse experiment. The trained DCNN then segments all images in the full time-lapse sequence (Fig. 1c, Supplementary Fig. 3). We provide open-source code for this fully automated image analysis approach enabling biologists to use DCNN-based segmentation in their routine live cell experiments (Supplementary Software). We also show that the general idea is applicable for other microscopy experiments where object-specific fluorescent stains can be used for definition of training regions.

## Results

### Experiment 1 - time-lapse bright-field sequence

The time-lapse dataset consisted of images from three wells, each image was sub-divided into nine sites, together containing approximately 200 cells. We used eight out of nine image sites from well 1 for training our DCNN and augmented the data by flipping and translations. The probability maps from the DCNNs had high values inside the cellular regions and low values towards the edges, and final segmentation results of individual cells were automatically generated by watershed-based segmentation using CellProfiler.

We evaluated the segmentation results by comparing to manual as well as automatically generated ground truth. The creation of manual ground truth was tedious and challenging and we found it difficult to delineate the cell boundaries of clustered cells and cells undergoing cell division. The automatically generated ground truth was based on fluorescence data, using standard segmentation functions in CellProfiler. Identifying touching cells was comparatively easier for the fluorescence data since we had a nuclear stain and nuclei could be identified as distinct structures even though the cells were touching. We observed that this was a source of error when we compared to the manual ground truth based on bright-field data.

**Figure 2:**
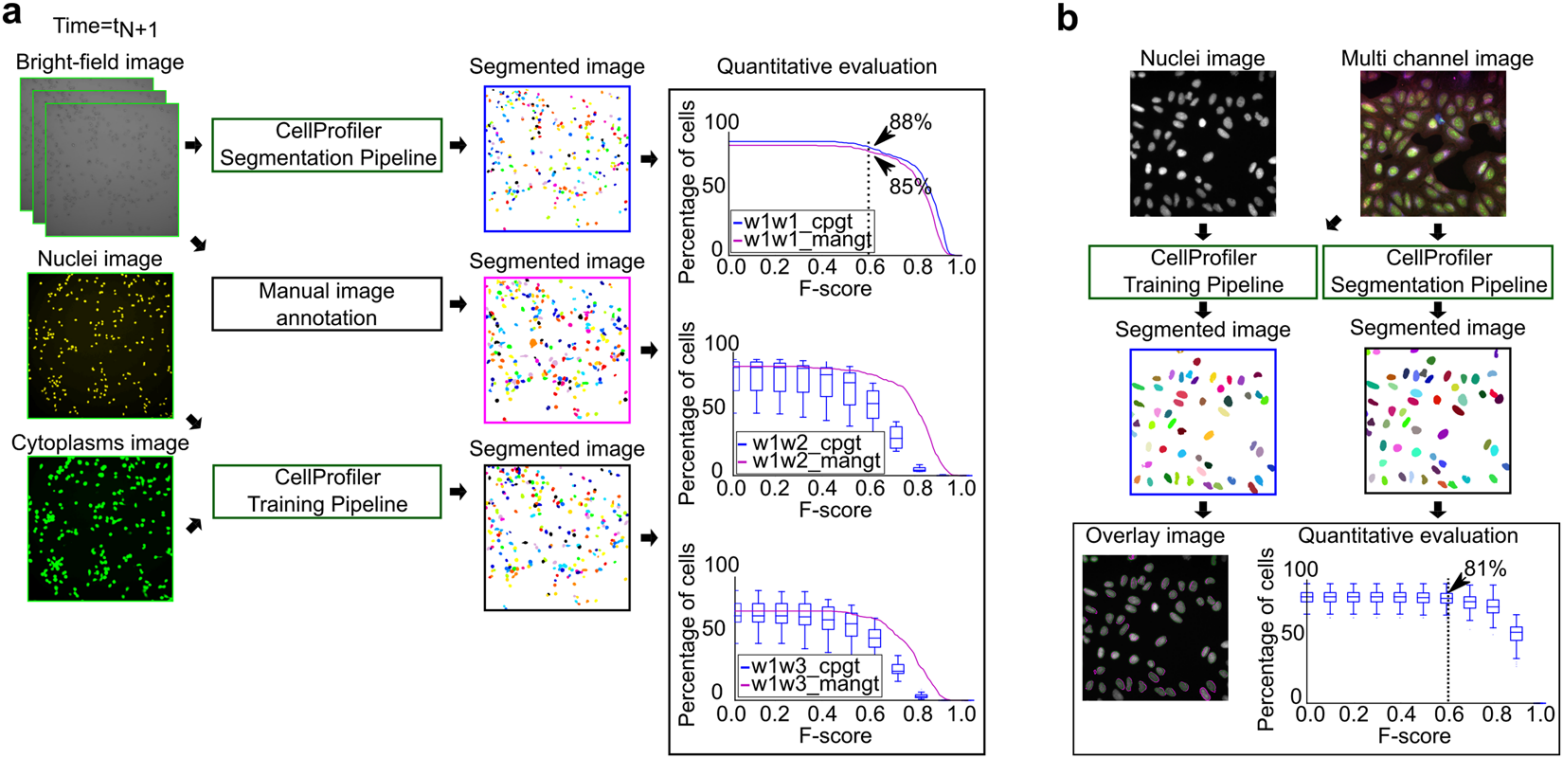
Segmentation evaluation pipelines. (**a**) In experiment 1, the previously un-seen bright-field channel of the test image was fed to the CellProfiler segmentation pipeline containing the trained DCNN. The same bright-field channel was manually annotated to create manual ground truth (‘mangt’) for comparison. The parallel fluorescent channels corresponding to the bright-field channel was fed to the CellProfiler training pipeline to create automatic ground truths for evaluation (‘cpgt’). Note that the ground truth generated for evaluation was not used for training. The three plots show the percentage of cells segmented with an F-score greater than or equal to the F-score value shown in the horizontal axis. ‘w1w1_cpgt’ corresponds to the result obtained when the DCNN was trained on eight sites of well 1 and tested on the ninth site of well 1 with CellProfiler created ground truth (blue line plot), while ‘w1w1_mangt’ corresponds to a comparison with with manual ground truth (magenta line plot). The plot shows that 88% of the cells were segmented with an F-score ≥ 0.6 when compared with automatic ground truth while 85% of the cells were segmented with an F-score ≥ 0.6 when compared with manual ground truth. The second plot shows the result when the DCNN was trained on well 1 and tested on well 2. The magenta plot shows the comparison done on the single manually annotated image site while the blue box plot shows the result when compared with all the nine image sites in well 2. Similarly, the third plot corresponds to the result when the DCNN was trained on well 1 and tested on well 3. (**b**) In experiment 2, a four-channel input image and the nuclei channel were used to create the ground truth for training the DCNN. The trained DCNN was used to segment the four-channel test image. The result of the segmentation from the CellProfiler pipelines for creating ground truth and the segmentation pipeline using DCNN is overlaid on the nuclei image. The quantitative evaluation shows the percentage of cells greater than or equal to a particular threshold v/s the corresponding F-score values. The box plot shows when the comparison was done for 58 images. The median value shows that around 81% of cells were segmented with F-score ≥ 0.6.

A DCNN trained on eight sites of well 1 was quantitatively evaluated on the ninth site of well 1 (top plot) and on all nine unseen sites of wells 2 (center plot) and 3 (bottom plot in Fig. 2a). The horizontal axis of all three plots shows the threshold value (from 0 to 1) for the F-score (as described in the Supplementary method), while the vertical axis shows the percentage of cells with an F-score greater than or equal to the F-score threshold. For the evaluation on wells 2 and 3, the magenta lines correspond to comparison with manual ground truth on a single image site (‘w1w2_mangt’ and ‘w1w3_mangt’) while the blue box plots correspond to comparisons with CellProfiler-generated ground truth for each of the nine image sites (‘w1w2_cpgt’ and ‘w1w3_cpgt’). Segmentation results of some image sites in wells 2 and 3 lead to the low minimum value in the box plots. We believe this is due to cell population variations that are difficult to model with a different well during training. This suggests that a separate DCNN may be required for each well for optimal segmentation results. Using the proposed automatic ground truth generation and training a separate DCNN for each well we can overcome these inter-well variations with very limited manual input.

Two sample videos showing the segmentation performance are provided as Supplementary videos 1 and 2. Video 1 shows visually excellent results while video 2 illustrates some of the method’s limitations. We also provide the segmentation results overlaid on the input image in Supplementary Fig. 4.

### Experiment 2 - multi-channel fluorescent dataset

To further evaluate the method, we performed a second experiment segmenting cell nuclei from non-nuclei markers. Leaving out the specific stain targeting nuclei leaves the image channel to be used to image a different cellular structure. In this study, we used the image set BBBC022v1^9^, available from the Broad Bioimage Benchmark Collection^10^. Each site in this dataset was imaged at five different wavelengths distinguishing different intracellular structures such as nucleus, endoplasmic reticulum, nucleoli, golgi apparatus, plasma membrane, and mitochondria (Supplementary Fig. 5). Similar to the time-lapse experiment we created the ground truth automatically using CellProfiler. We used the nuclear channel to create the ground truth. The remaining four channels were used as data for training our DCNN. One image set is shown in Fig. 2b as multi-channel image and nuclei image. After creating the training set we trained our DCNN and the trained DCNN was used to segment the test set using the CellProfiler segmentation pipeline (Supplementary Software). We quantitatively and qualitatively evaluated the segmentation of 58 test images, and 81% of the cells have an F-score greater than or equal to 0.6 on an average (Fig. 2, Supplementary Fig. 6).

## Discussion

Deep learning is widely accepted as the state-of-the-art methodology in many automated data analysis tasks. Training and properly using DCNNs require domain knowledge and it is often necessary to tune several hyper-parameters for performance optimization. In addition, high-quality ground truth, which is often expensive to generate, is needed to train the network. Cell samples are diverse in nature and a new ground truth may be needed for each new sample. This creates a reluctance in the biology community to adopt the DCNN technology in routine cell analysis. In this work, we presented an approach to simplify the DCNN training by creating an automated image analysis pipeline for generation of ground truth. We quantitatively evaluated the segmentation results and showed that the DCNN trained on the automatically created ground truth worked similar to manual annotations. We implemented the entire image analysis for creating the ground truth for training the DCNNs and segmenting the cell images as a set of CellProfiler pipelines so that biologists can readily use them in practise.

## Methods

### Cell culturing and image acquisition for experiment 1 (time-lapse)

Human lung carcinoma cells (H299) were seeded to low density on a glass bottom 96 well-plate (MoBiTec #5241-20) and cultured for 6 h in RPMI medium (RPMI 1640, Gibco #42401) supplemented with 10% Fetal Bovine Serum (Invitrogen, #10270-106). 30 min before the start of recording, the culture medium was exchanged to fresh warm medium (300 *μ*l/well) to remove floating debris. Images were acquired using the NIS software and a 10x/0.45 air objective on an inverted Nikon Ti microscope, motorized in xy and z (stage piezo, Mad City Labs) and equipped with a full size incubator (Live Imaging Service), a Halogen lamp as light source for bright-field light imaging, LEDs (SpectraX, Lumencor) as light source for fluorescence imaging (Hoechst: ex 395/25; Cell Mask: ex 475/28), an external filter wheel for emission filters (Hoechst: em 447/60; Cell Mask: em 500LP) and an Andor Zyla 4.2+ camera. To help the repositioning of the plate after labeling the cells at the end of the time-lapse acquisition, the plate was pushed to the top right corner of the stage insert and the lid was then placed on top without moving the plate. The microscope optics were aligned (Koelhlering) prior to imaging and only the center of the well was imaged in order to minimize distortions at the edges of the wells in the bright-field image.

Bright-field images were recorded every 10 min overnight (total imaging time 17 h) until the recording was stopped in the morning immediately before the final staining and imaging steps. Images from 9 sites from the center of each well were acquired. The focus was maintained using the Nikon Perfect Focus System set to focusing where the filopodia at the edges of the cells were best contrasted and a z-stack of 2 additional z-planes upwards (z-step 4 *μ*m) was acquired for each image. All images are of size 2034 × 2034 pixels.

In the morning, the acquisition was stopped, the lid of the plate removed and 150 *μ*l of medium from each well were vortexed with 1 *μ*l of blue nuclear dye and 1 *μ*l of green cytoplasmic dye (Hoechst 33342, Invitrogen, CellTracker CMFDA, #C7025, reconstituted according to the manufacturer’s instructions) and added back to each well. The plate was repositioned, the lid placed on top and after a total of 10 min after the end of the time-lapse, images in bright-field as well as blue and green fluorescence were acquired at the same positions and with the same z-stack to serve as input for creation of ground truth.

Finally the dataset consisted of images from three micro-wells. Only the last 44 time-points of the bright field time-lapse sequence were used for data analysis as shown in Fig. 1a. Previous time points were discarded. The starting time of imaging is shown as *t*_1_ and the end of the sequence is shown as *t*_*N*_. The bright-field image and two fluorescent images captured after staining also consist of three focus levels per image as shown in Fig. 1b. The time *t*_*N*+1_ shows that the images were acquired after staining.

### Input data for experiment 2

We used images of human U2OS cells from a compound-profiling cell painting experiment openly available from Broad Bioimage Benchmark Collection (BBBC) with accession number BBBC022v1. The selected subset from the BBBC dataset contained 522 images from 9 sites of 58 wells from a single experiment. One site per well was used for testing and the remaining 8 images from all the wells were pooled to create the training set. Each site in this dataset was imaged at five different wavelengths distinguishing different intracellular structures such as nucleus, endoplasmic reticulum, nucleoli, golgi apparatus plasma membrane, and mitochondria (Supplementary Fig. 5). All images in the BBBC dataset were of size 696 × 520 pixels in the horizontal and vertical directions respectively.

### Automatic training set generation

Automatic training set generation was one of the key steps in the whole analysis process. We used CellProfiler^7^, an openly available software, to create the training set automatically. To create the training set we used the z-stack bright-field, fluorescent nuclei and cytoplasms images acquired after staining as shown in Fig. 1b. We used eight out of the nine sites for creating the training set and one image to create an independent evaluation set. The bright-field images were used to create the training data and both the fluorescent nuclei and cytoplasms channels were used to create the ground truth labels. The non-uniform illumination of the input bright-field images were corrected by an iterative spline fitting^11^ (Fig. 1a,b). We applied illumination correction separately on all the z-stacks.

To create the ground truth labels we took the fluorescent nuclei channel and corrected the illumination variations separately on the three slices of the z-stacks. After the illumination correction we average projected the z-stacks to create a single channel image for further processing. This process was repeated to the fluorescent cytoplasms channel also. Thereafter we segmented the nuclear regions from the nuclei channel by global thresholding followed by watershed segmentation. Next we took the single channel cytoplasms image and enhanced the contrast by grayscale dilation with a disk-shaped structuring element of diameter 5 pixels. After the dilation, we used the nuclear regions as seeds and applied a seeded watershed segmentation on the dilated cytoplasms image to get the final segmentation mask of the cells (Fig. 1b) We repeated the above process on all the three datasets using the same CellProfiler pipeline provided as Supplementary Software.

For the BBBC dataset we followed a similar approach as we did for the time-lapse dataset. We illumination corrected all the images from the five channels separately. After the illumination correction we used the nuclei channel for creating ground truth and the remaining four channels were used as data for training. To create the ground truth we thresholded the nuclei image by global thresholding followed by a watershed segmentation. The pipeline for creating the training set is provided as part of the Supplementary software.

### Manual ground truth generation for evaluation

In addition to the automatic ground truth we also created manual annotations to evaluate the performance of our network trained on automatically generated ground truth for the time-lapse dataset. We manually annotated one image, unused in training set creation, out of the nine images. We found that each image contained approximately 200 cells. Manual annotation took around two hours for a single image. We created manual annotations for all the three wells. The manually generated ground truth was only used to evaluate the final segmentation performance of the DCNN and not for training the network.

### Deep convolutional neural network architecture

We used a fully convolutional neural network approach for creating our DCNN architecture. In DCNNs, usually one layer in the neural network is connected to another layer that immediately follows it. If we bypass one or more layers in the network and connect to a subsequent layer, then it is known as a skip connection. Recently it was showed that residual connections or short skip connections improved classification performance on natural images^12^. Ronneberger *et al*.^3^ showed that long skip connections can improve segmentation of biological images. Recently, Drozdzal *et al*.^13^ showed that both long and short skip connections gave better performance in biological applications. We used both long and short skip connections for our network. The network architecture is as shown in Supplementary Fig. 2a.

We used batch normalization^14^ as regularization after every convolution. We used the MSRA method^15^ to initialize the weights in the convolutional layers and set the input image size to 480 × 480 pixels. The first layer had 8 feature maps and doubled the feature map size with every down-sampling. The long skip connections from the downward path were element-wise summed and batch normalized at the receiving end of the upward path as shown in Supplementary Fig. 2a as right pointed arrows. We used a weighted softmax loss function.

We used four building blocks to create the entire network structure. We named the blocks as Conv, DeConv, Conv-Pool, and Residual. The different blocks used are shown in Supplementary Fig. 2b. The Conv block contained a convolution layer followed by batch normalization and rectified linear unit^16^ (ReLU) activation function. We used the Conv block in other blocks also. The DeConv block contained one deconvolution followed by two Conv blocks. The deconvolution layer up-sampled the feature maps. The Conv-Pool block contained one convolution followed by batch normalization, ReLU, and pooling. In the pooling layer we used maximum pooling with a pool size of 2 × 2 and stride of 2 × 2. The feature map size was reduced to half its original size in the vertical and horizontal directions after the pool layer. The Residual block contained three Conv blocks. The first Conv block generated 16 feature maps using 1 × 1 convolution; the second block generated 16 feature maps using 3 × 3 convolutions; and the third block generated 64 feature maps using 1 × 1 convolutions. We added the input and the output from the third Conv block in an element-wise manner. The result of the addition was batch normalized and formed the final output of the Residual block. The same DCNN architecture was used in both experiment 1 and 2. We provide the network architecture in a text file as part of the Supplementary Software.

### Data preprocessing

For the DCNN training we used eight out of the nine image sites in our training set and kept one image for evaluation. The data preprocessing step involved processing both the input bright-field images and the ground truth labels created automatically by our image processing pipeline in CellProfiler (Supplementary Software). We augmented the image data by spatial transformations to increase the dataset size as illustrated in Fig. 1b. Both the input bright-field images and the corresponding label images were augmented to maintain spatial consistency. The data augmentation step consisted of random flipping in left-right and up-down directions followed by the creation of an image that was nine times the input image by extending the image on all sides by mirroring. From the extended image, we randomly cropped image patches of size 480 × 480 pixels. After cropping we normalized the bright-field images to a range between 0 and 1 and subtracted the median value from the image.

There were typically fewer foreground than background pixels in our dataset. Such imbalance in the dataset biases the network towards the background rather than the foreground. To compensate for this imbalance we created a weighted label image, in addition to the label image. We set the weight in such a way that the weighted sum of the number of pixels belonging to one class was the same as that for the other class. This enabled the loss function to treat the foreground and background regions with equal importance. We forced the network to learn the features corresponding to the foreground regions by setting an additional weight of 3 to the foreground region. The choice of this parameter depended on the dataset, based on the density of cells in the image, and could be automatically determined based on the proportion of cells covering the training image. The entire pipeline for the data preprocessing is provided as a Python program as part of the Supplementary Software.

### DCNN training

After data augmentation our training set consisted of 6000 images saved in HDF5 data format. We set the initial DCNN learning rate to 0.001, trained the network for 60000 iterations and reduced the learning rate to 1/10 of the current value after every 5000 iterations. We used RMSProp^17^ optimization and the open source tool Caffe^18^ to train our DCNN. We used the same amount of training data and the hyper-parameters for both our experiments. The DCNN configuration and hyper-parameters are provided in a text file as part of the Supplementary Software.

### Cell segmentation

The input images were preprocessed similarly to the training set. The input images were too large to process using our DCNN due to the limitations in the size of GPU memory. We therefore divided the images into non-overlapping tiles of size 240 × 240. We observed that the probability maps output by the DCNN had errors at the edges of the tiles (Supplementary Fig. 7). To solve this, we performed a second pass through the image after shifting the tile positions to half the tile size, i.e., 120 pixels to the left and up compared to the initial pass. The shifting centered the new tile at the position where there was an edge in the first pass, resulting in two probability maps for the same image. We created a Gaussian weighted image that gave high values to the central region and low values to the tile edges for the first probability map. Similarly, we created another Gaussian weighted image for the second probability map. Then we multiplied the probability maps with the corresponding Gaussian weighted images and summed the result to get a final probability map without edge artifacts (Supplementary Software). The workflow to create probability maps without tiling artifacts is shown in Supplementary Fig. 7. The bright regions in the output probability map correspond to the detected cell regions. We used the watershed segmentation algorithm in the CellProfiler software to convert the final probability maps to segmentation masks. Our DCNN consistently under-estimated the extent of the cells. To compensate for this we postprocessed the final segmentation masks by dilation with a structuring element of size 9 × 9 for the time-lapse sequence dataset and 3 × 3 for the BBBC dataset. The final segmentation mask with pseudo coloring of the segmented cells is shown in Supplementary Fig. 3 iii. A segmentation result overlaid on the bright-field image is shown in Supplementary Fig. 4. The entire cell segmentation is provided as CellProfiler pipelines, as part of the Supplementary Software.

### DCNN system

The DCNN system was set up on a workstation with six core Intel(R) Core(TM) i7 CPU running at 3.5 GHz and 32 Gb RAM equipped with a Nvidia Titan X Pascal GPU on Ubuntu 14.04 operating system. The training of a single DCNN took nearly 5.5 hours. It took nearly 6 seconds to segment a single time-lapse image of size 2034 × 2034 including pre- and postprocessing.

### Software availability

The software for data preprocessing, training and evaluation set creation and cell segmentation are implemented as CellProfiler pipelines and is available as part of Supplementary Software. The program for data augmentation is provided as a Python script. The DCNN architecture and the configuration files are provided as text files. The trained neural network model is also provided as a caffe model as part of Supplementary Software. The details on installation and configuration of the Supplementary Software is provided as a Supplementary note.

### Statistics and analysis

The minimum, maximum and median F-score value for each segmented cell region were calculated in Python and visualized using matplotlib^19^. The data augmentation for training the neural network was done using Python. The automatic ground truth creation was done as a CellProfiler pipeline.

### Data availability statement

The time-lapse dataset that was used in this study is availble at http://cb.uu.se/∼carolina/timelapse_data/. The BBBC dataset available from the Broad Bioimage Benchmark Collection under accession code BBBC022v1.

## Electronic supplementary material

supplementary

Supplementary video 1

Supplementary video 2

## References

[1] Meijering E (2012). Cell segmentation: 50 years down the road [life sciences]. IEEE Signal Proc. Mag..

[2] LeCun Y, Bengio Y, Hinton G (2015). Deep learning. Nature.

[3] Ronneberger, O., Fischer, P. & Brox, T. U-net: Convolutional networks for biomedical image segmentation. In *International Conference on Medical Image Computing and Computer-Assisted Intervention*, 234–241 (Springer, 2015).

[4] Ciresan, D., Giusti, A., Gambardella, L. M. & Schmidhuber, J. Deep neural networks segment neuronal membranes in electron microscopy images. In *Advances in neural information processing systems*, 2843–2851 (2012).

[5] Van Valen DA (2016). Deep learning automates the quantitative analysis of individual cells in live-cell imaging experiments. PLoS Comput Biol.

[6] Eulenberg, P. *et al*. Deep learning for imaging flow cytometry: Cell cycle analysis of jurkat cells. *bioRxiv* 081364 (2016).

[7] Carpenter AE, Jones TR, Lamprecht MR (2006). Cellprofiler: image analysis software for identifying and quantifying cell phenotypes. Genome Biology.

[8] Liimatainen, K., Ruusuvuori, P., Latonen, L. & Huttunen, H. Supervised method for cell counting from bright field focus stacks. In *Biomedical Imaging (ISBI), 2016 IEEE 13th International Symposium on* 391–394 (IEEE, 2016).

[9] Gustafsdottir SM (2013). Multiplex cytological profiling assay to measure diverse cellular states. PloS one.

[10] Ljosa V, Sokolnicki KL, Carpenter AE (2012). Annotated high-throughput microscopy image sets for validation. Nat Methods.

[11] Lindblad, J. & Bengtsson, E. A comparison of methods for estimation of intensity non uniformities in 2d and 3d microscope images of fluorescence stained cells. In *Proceedings of the Scandinavian Conference On Image Analysis* 264–271 (2001).

[12] He, K., Zhang, X., Ren, S. & Sun, J. Deep residual learning for image recognition. In *Proceedings of the IEEE Conference on Computer Vision and Pattern Recognition* 770–778 (2016).

[13] Drozdzal, M., Vorontsov, E., Chartrand, G., Kadoury, S. & Pal, C. The importance of skip connections in biomedical image segmentation. In *International Workshop on Large-Scale Annotation of Biomedical Data and Expert Label Synthesis* 179–187 (Springer, 2016).

[14] Ioffe, S. & Szegedy, C. Batch normalization: Accelerating deep network training by reducing internal covariate shift. In *Proceedings of The 32nd International Conference on Machine Learning* 448–456 (2015).

[15] He, K., Zhang, X., Ren, S. & Sun, J. Delving deep into rectifiers: Surpassing human-level performance on imagenet classification. In *Proceedings of the IEEE international conference on computer vision* 1026–1034 (2015).

[16] Nair, V. & Hinton, G. E. Rectified linear units improve restricted boltzmann machines. In *Proceedings of the 27th international conference on machine learning (ICML-10)* 807–814 (2010).

[17] Tieleman, T. & Hinton, G. Lecture 6.5—rmsprop: Divide the gradient by a running average of its recent magnitude. COURSERA: Neural Networks for Machine Learning (2012).

[18] Jia, Y. *et al*. Caffe: Convolutional architecture for fast feature embedding. In *Proceedings of the 22nd ACM international conference on Multimedia* 675–678 (ACM, 2014).

[19] Hunter JD (2007). Matplotlib: A 2d graphics environment. Computing In Science & Engineering.

