# Supplementary material for: Automated Training of Deep Convolutional Neural Networks for Cell Segmentation
Source: Sci Rep. 2017 Aug 10;7:7860. doi: 10.1038/s41598-017-07599-6 (PMC5552800; doi:10.1038/s41598-017-07599-6)

Automated Training of Deep Convolutional Neural Networks for Cell Segmentation

Sajith Kecheril Sadanandan, Petter Ranefall, Sylvie Le Guyader and Carolina Wählby

Downloading the datasets	Instructions for finding the datasets online
Supplementary method	Evaluation of multi-object segmentation using the F-score
Supplementary Figure 1	Multi focal time-lapse data for training
Supplementary Figure 2	The DCNN architecture
Supplementary Figure 3	Time-lapse sequence segmentation
Supplementary Figure 4	Segmentation result overlayed on time-lapse dataset
Supplementary Figure 5	Multi channel BBBC dataset for training
Supplementary Figure 6	Segmentation result overlayed on BBBC dataset
Supplementary Figure 7	Removing tiling artifacts from probability maps
Supplementary note	Supplementary software installation instructions
Supplementary videos 1 and 2	Segmentation result overlayed on bright-field time-lapse images
Supplementary software	CellProfiler, Caffe, CellProfiler-Caffe bridge,<br>(currently also available at<br>http://cb.uu.se/~carolina/timelapse_data/)

1 Downloading the datasets

The time-lapse dataset can be downloaded from
http://cb.uu.se/~carolina/timelapse_data/. The file *trainingdata.zip* (1Gb) contains the multi-channel fluorescent and the bright-field images used to create automatic ground truth. The file *sequence.zip* contains the multi-focal time-lapse bright-field images.

The BBBC dataset used in this study is available at
https://data.broadinstitute.org/bbbc/BBBC022/

Supplementary method

Evaluation of multi-object segmentation using the F-score

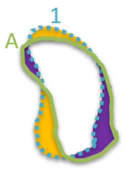

For the true object A (solid green line) we achieve the segmentation result 1 (dashed blue line). This means that we have True Positive pixels where A and 1 overlap (TP=white), False Positive pixels where 1 includes more than the true object (FP=yellow), and False Negative pixels where 1 misses part of the true object A (FN=purple).

We can then define
Accuracy=(TP)/(TP+FN+FP)
Recall (=Sensitivity)=TP/(TP+FN) *will ignore FP*
Precision=TP/(TP+FP) *will ignore FN*

Accuracy is often used as a performance measure, but does not provide good metric if the classes are unbalanced. More common is the the F-score (sometimes called F-factor or F1-measure), also referred to as a harmonic mean of recall and precision

F-score=2*precision*recall/(precision+recall)

The F-score is in fact the same thing as the popular Dice's coefficient very often used to evaluate segmentation results (where A and B in this case refer to two different outlines):
Dice's coefficient=2(A∩B)/(A+B) =2*TP/(FP+FN+2*TP)=F-score!!!

For multiple objects, as the case in the paper and the illustration below, we associate each detected object 1-5 with its most overlapping true reference object A-E. If the reference object is shared with another detected object, we associate the two objects with the most overlap. F-score (or Dice's coefficient) is then calculated on a per object basis.

1—A, F-score=0.80
2—none, F-score=0
3—B, F-score=0.60
4—D, F-score=0.65
5—none, F-score=0
C&E are false negatives, each with F-score=0

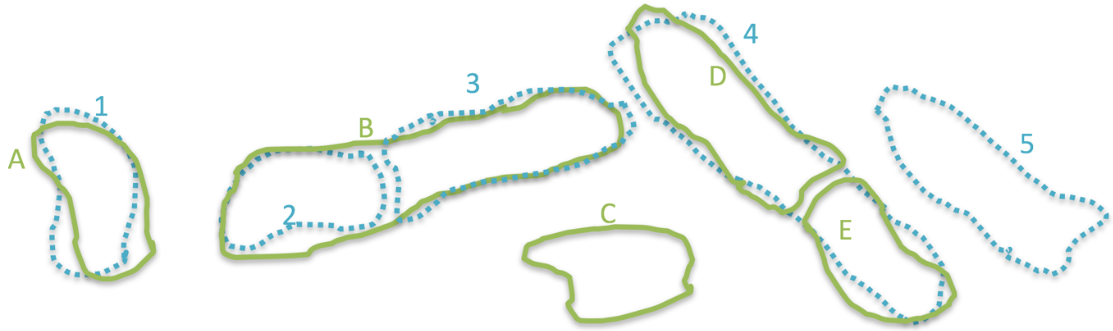

Supplementary figures

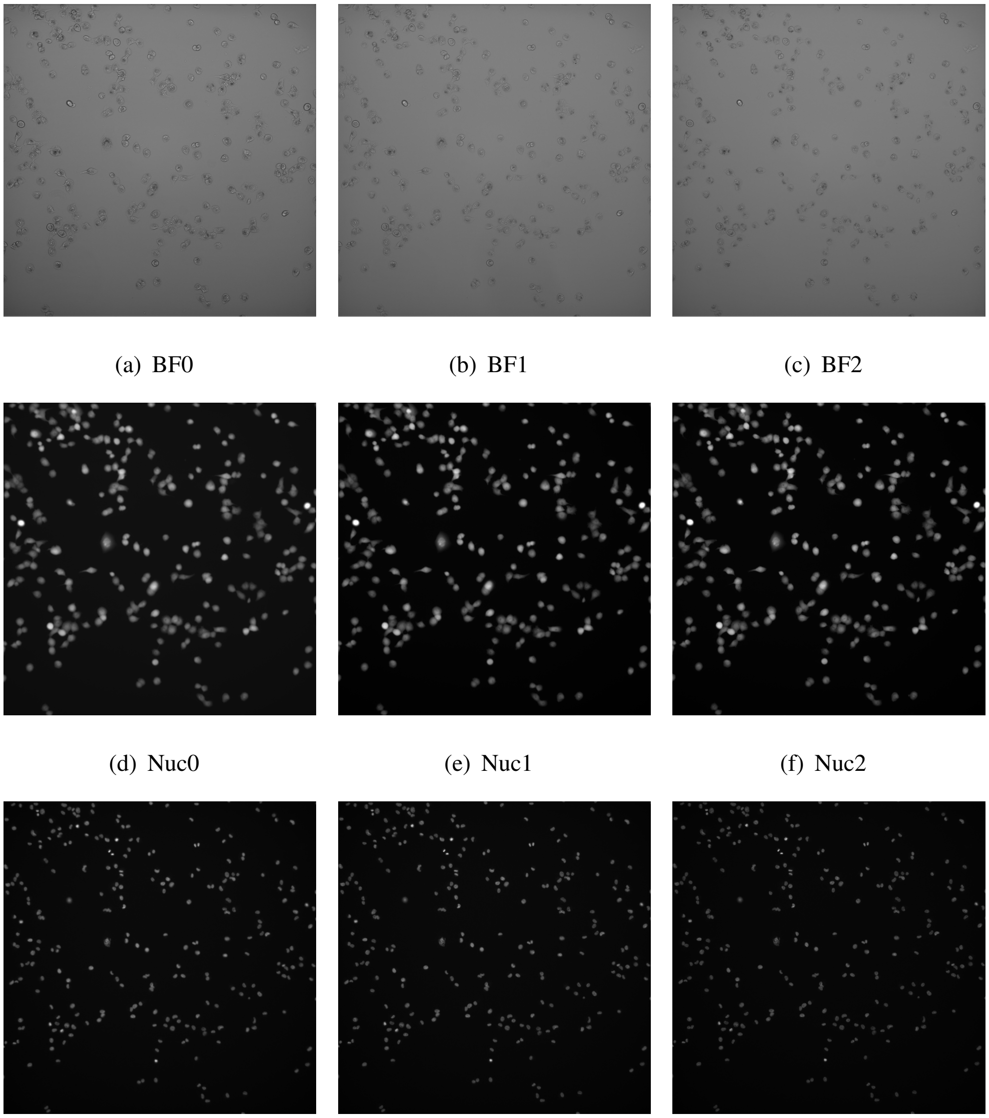

(a) BF0 (b) BF1 (c) BF2

(d) Nuc0 (e) Nuc1 (f) Nuc2

(g) Cyto0 (h) Cyto1 (i) Cyto2

Fig. 1. Multi focal time-lapse image dataset for training. The dataset consist of (a-c) bright-field, (d-f) fluorescent cytoplasm and (g-i) fluorescent nuclei for focus levels 1, 2 and 3 respectively.

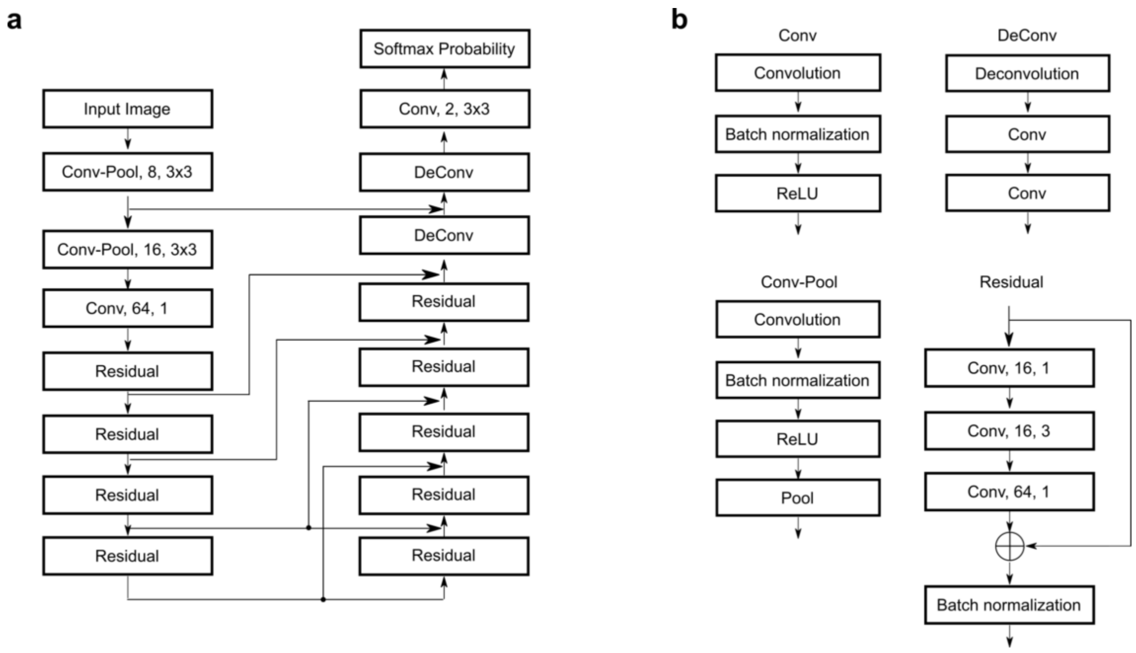

Fig. 2. The Deep Convolutional Neural Network (DCNN) architecture. (a) The overall network architecture. (b) The sub blocks used to create the network. The Conv block consists of a convolution layer followed by batch normalization and rectified linear unit activation. The DeConv block consists of one deconvolution or upsampling followed by two convolution layers. The Conv-Pool layer is similar to the Conv layer except that after ReLU the output is downsampled (max pooled). The Residual block consists of a shortcut connection from the input to the output that is summed element-wise followed by batch normalization.

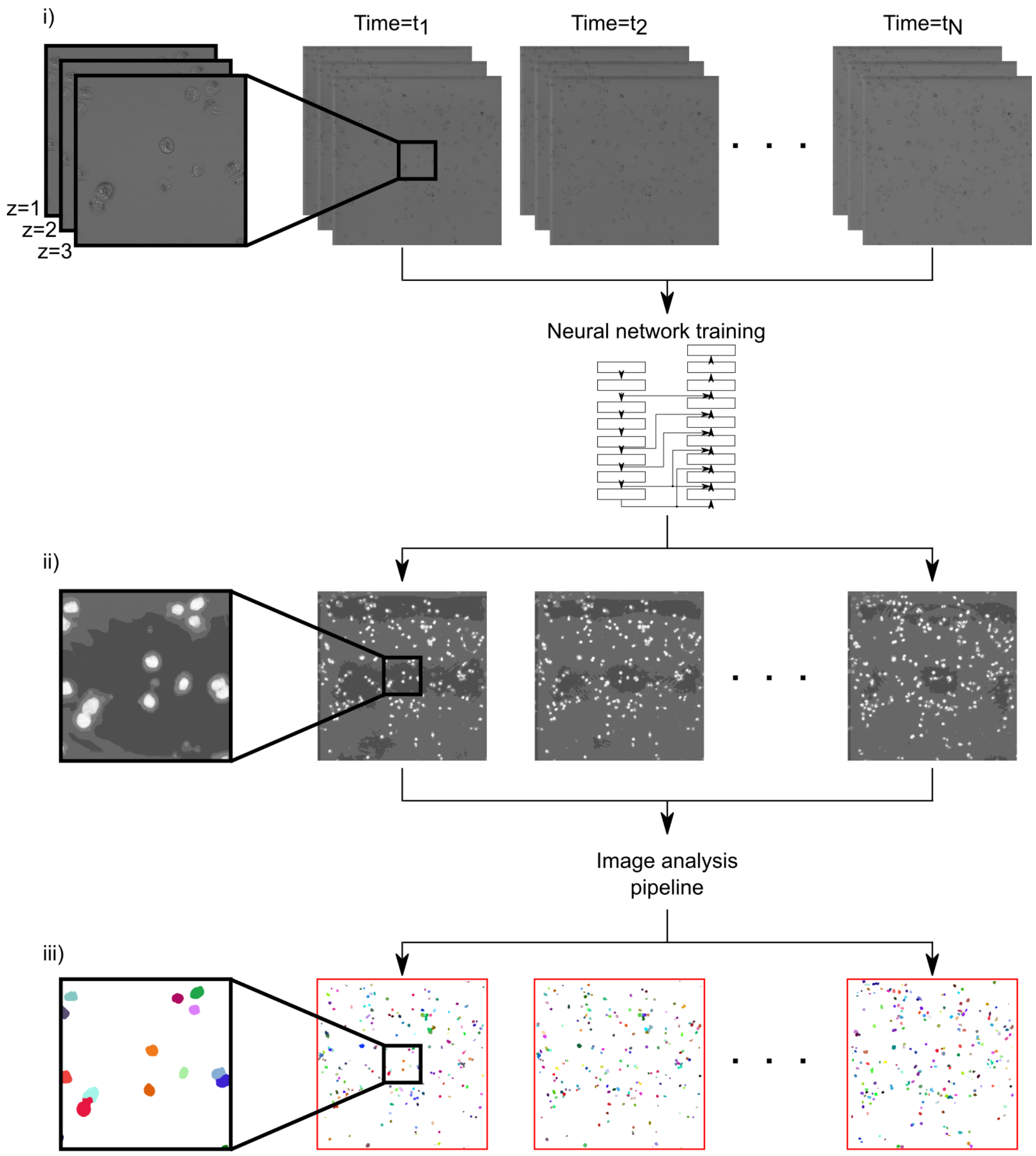

Fig. 3. Sequence segmentation. After training the network on the last time sample (t_{N+1}), the network is used to segment the whole time-lapse sequence ($t_1 \dots t_N$). (i) shows the input multi channel time-lapse images, (ii) shows the corresponding probability maps and (iii) the final segmented cell regions.

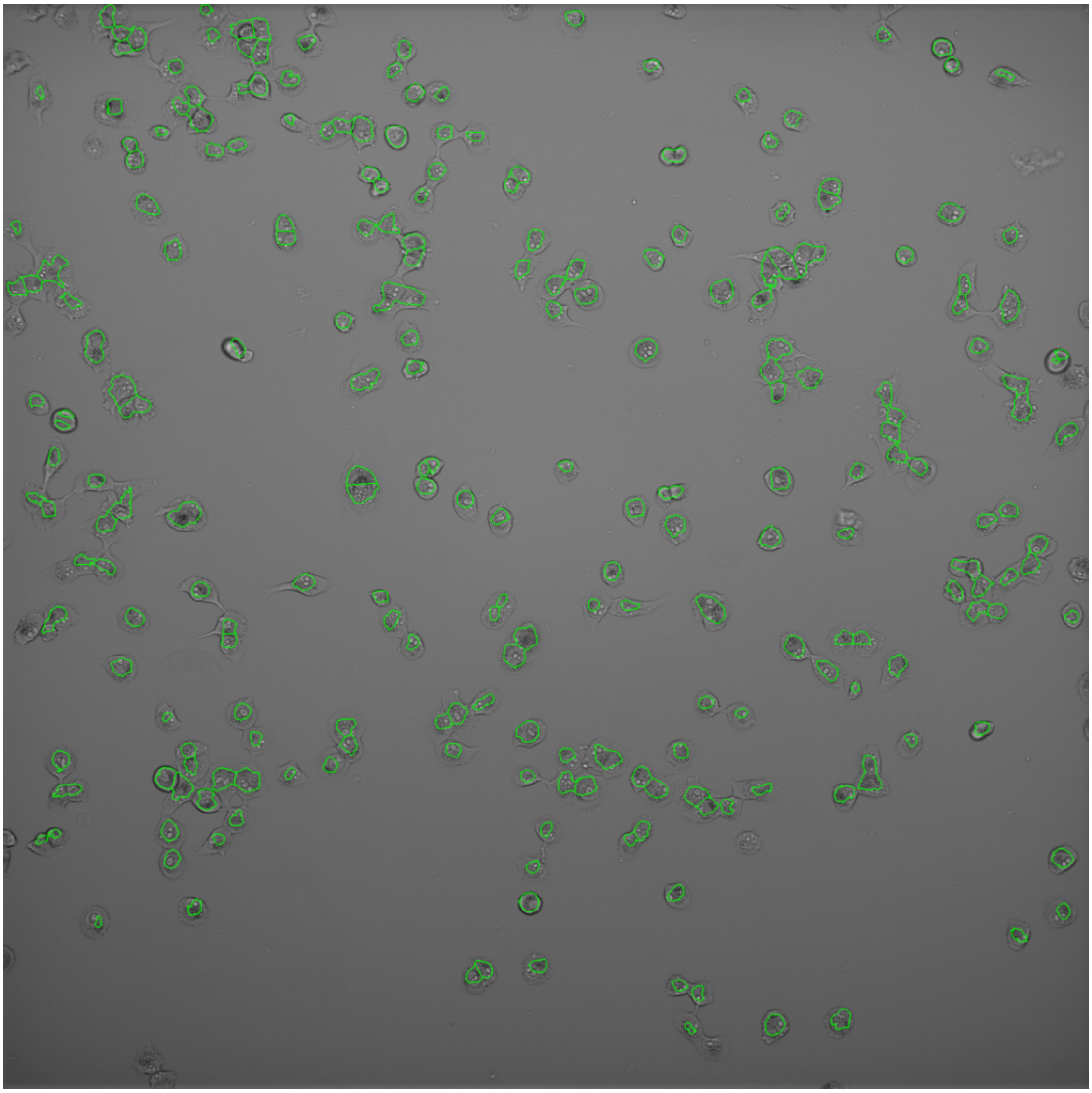

Fig. 4. Example image from the time-lapse dataset segmented by the DCNN. The result is overlayed (green) on the input bright-field image. For overlaying, only the first focus level is used.

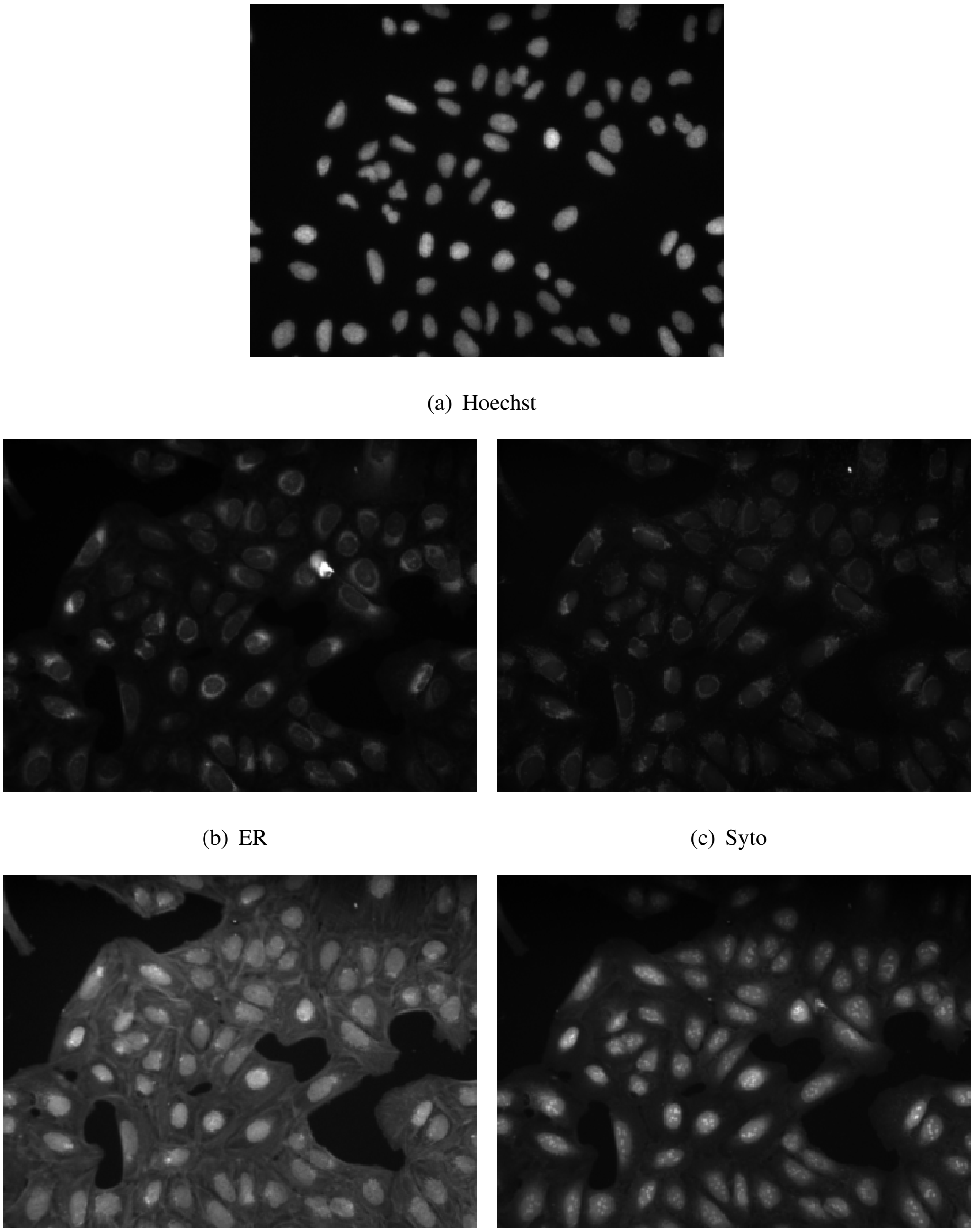

(a) Hoechst

(b) ER

(c) Syto

(d) Ph_golgi

(e) Mito

Fig. 5. Multi channel BBBC dataset for training. (a) Hoechst (b) Endoplasmic reticulum, (c) Syto, (d) Ph_golgi, and (e) Mitochondria labeled images. Image set (a) was used for creating automatic ground truth and images (b-e) were used as data for training. After training the neural network we detect the nuclear regions using the image set (b-e).

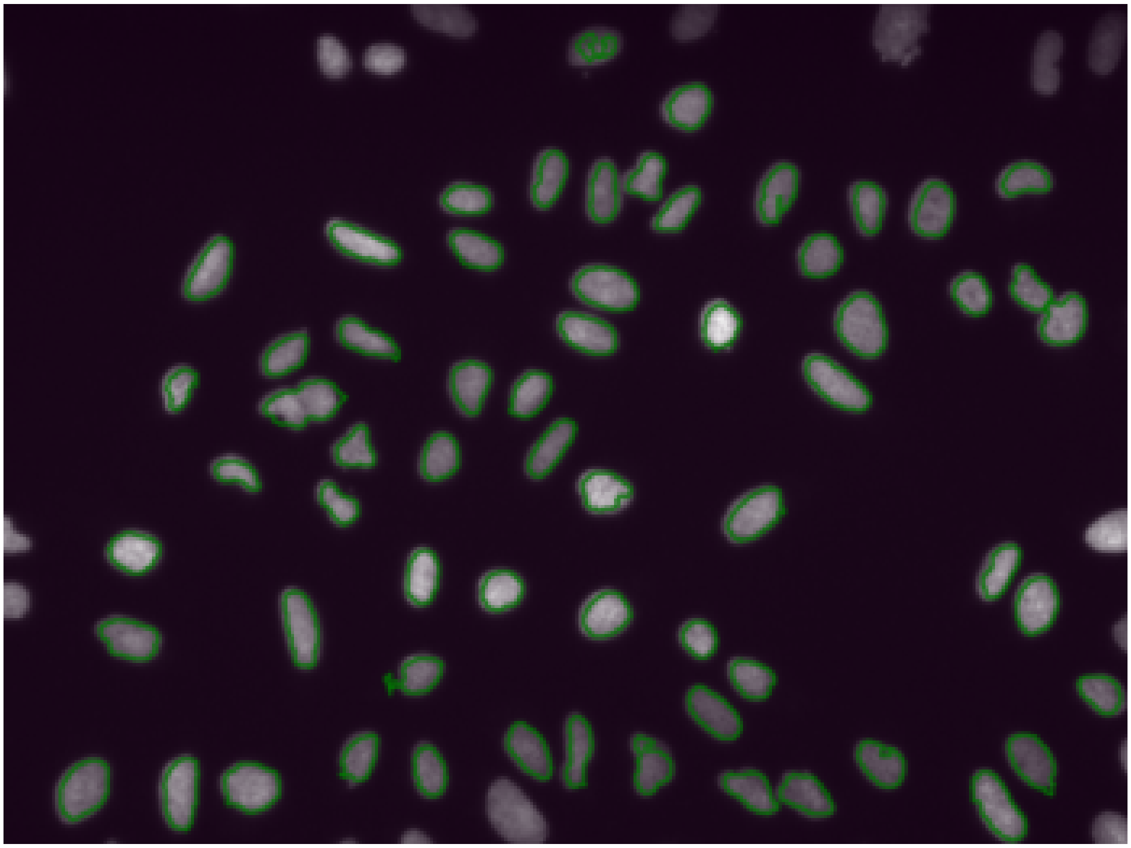

Fig. 6. Sample image from BBBC dataset segmented by the DCNN. The output is overlayed (green) on the Hoechst channel. The objects touching the image borders are removed.

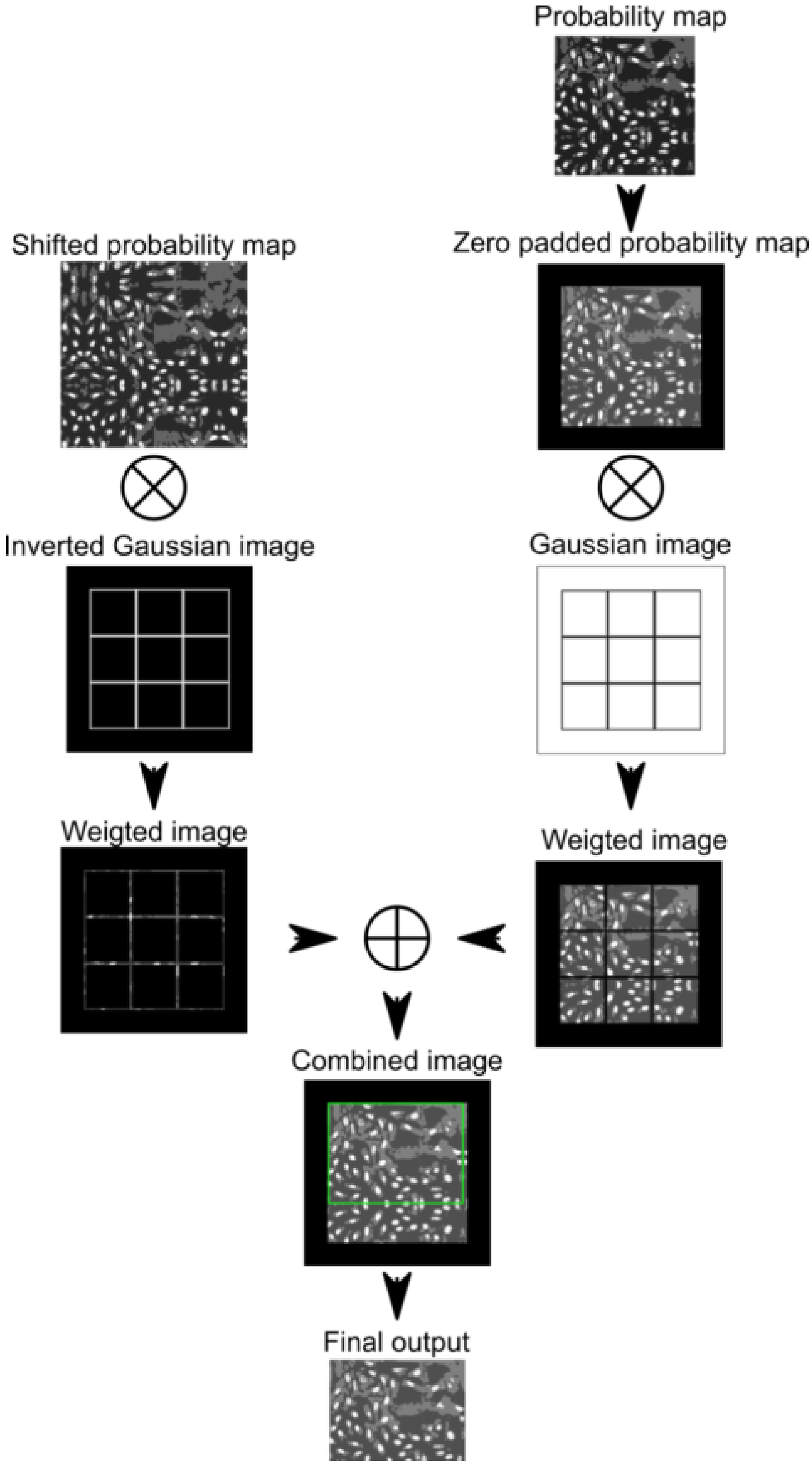

Fig. 7. The work flow to create probability map without tiling artifacts.

Supplementary note: Supplementary software installation instructions

1 Folder structure

```
software
├── caffe
├── caffe_models
│   ├── bbbc
│   │   └── protofiles
│   └── ki
│       ├── protofiles
│       ├── well1
│       ├── well2
│       └── well3
├── CellProfiler
├── cp_pipelines
│   ├── bbbc
│   └── ki
├── cp_plugin
└── python_files
```

2 Caffe installation on Ubuntu 14.04

1. Install the dependencies mentioned at `http://caffe.berkeleyvision.org/install_apt.html`
   (a) sudo apt-get install libprotobuf-dev libleveldb-dev libsnappy-dev libopencv-dev libhdf5-serial-dev protobuf-compiler
   (b) sudo apt-get install –no-install-recommends libboost-all-dev
   (c) sudo apt-get install libgflags-dev libgoogle-glog-dev liblmdb-dev
   (d) sudo apt-get install libopenblas-dev
   (e) sudo apt-get install python-opencv
   (f) sudo pip install protobuf
   (g) sudo apt-get install python-pywt

2. Go to *software/caffe*
   (a) Edit the **makefile.config** for enabling/disabling gpu support etc.
3. Continue with compilation.
   (a) make all
   (b) make pycaffe

3 CellProfiler installation

1. Install the dependencies for CellProfiler mentioned at `https://github.com/CellProfiler/CellProfiler/wiki/Source-Installation-(Ubuntu-14.04-LTS)` such as the following
   (a) sudo apt-get update
   (b) sudo apt-get upgrade
   (c) sudo apt-get install cython git libmysqlclient-dev libhdf5-dev libxml2-dev libxslt1-dev openjdk-7-jdk python-dev python-pip python-h5py python-matplotlib python-mysqldb python-scipy python-vigra python-wxgtk2.8 python-zmq
   (d) sudo pip install –upgrade cython
   (e) sudo pip install dask
   (f) sudo pip install –upgrade six
2. Go to the folder *software/CellProfiler*
   (a) sudo pip install –editable . –process-dependency-links
   (b) sudo pip uninstall prokaryote
   (c) sudo pip install prokaryote
   (d) sudo pip install javabridge
3. Go to the folder *software/cp_plugin* and edit the file **CaffePixelClassifier.py** in
   (a) Uncomment line 409 if CPU is used
   (b) Uncomment lines 406 to 408 if GPU is used
4. In 'Terminal'go to the folder *software/CellProfiler*
   (a) Run CellProfiler by command

   `python CellProfiler.py`

   (b) In CellProfiler, go to File → Preferences → CellProfiler plugins directory, browse to the location *software/cp_plugin* and click OK.
   (c) Close and reopen the CellProfiler to make the new changes

4 Other dependencies

1. Dependencies for python pipelines
   (a) sudo pip install tifffile
   (b) sudo pip install progressbar

5 Instructions on automatic training of deep convolutional neural network

1. Create the training set
   (a) Run the CellProfiler pipeline to create the required training set. ex. **bbbcTrainsetCreatePipeline.cpproj**. Configure the input files and output locations.

2. Data augmentation of training set
   (a) Go to *software/python_files* open the required file for data augmentation ex. **bbbcDataAugment.py**.
   (b) Edit lines
      i. 157 to add input image location
      ii. 158 to add segmented image location
      iii. 159 to add output folder location
      iv. 169 to add the number of images per file
      v. 170 to add the number of files
      vi. Save and run the file using command
      `python bbbcDataAugment.py`

3. Training the network model
   (a) Go to the output folder location for the dataset files created in the previous step 2((b))iii and copy the location of the file named **train.txt**, which contains the location of all the hdf5 files created.
   (b) Go to *software/caffe_models/bbbc/protofiles*
      i. Open the file **trainbbbc.prototxt**
      ii. In line 3, at the variable 'source' paste the location copied in step 3.a. ex.'/<location of dataset>/train.txt'. Save and close the file
      iii. Open the file **solver.prototxt**
      iv. In line 1, add the location of network file ex. '/<location of training file>/trainbbbc.prototxt'
      v. In line 11, add the location where network model to be created. Ex. '/<location of network model/model>'

vi. In line 12, add CPU/GPU for the respective hardware for training. (Training on GPU is usually two orders of magnitude faster than training on CPU)

vii. Save and close the file

(c) Open ‘Terminal’ and go to *software/caffe*

i. Run the command

```
./build/tools/caffe train -solver=<solver file location>/solver.prototxt
```

6 Cell segmentation using the deep convolutional neural network

1. Open CellProfiler

2. Load the required pipeline from the corresponding folders in *software/cp_pipelines* ex. **bbbc/bbbcEvalsetSegmentationPipeline.cpproj**

3. Follow the instructions on configuring the pipeline specified in section Configuring CellProfiler pipelines below and run the pipeline

Configuring CellProfiler pipelines

bbbcExtractSubset.cpproj

Images: Drag-and-drop your images to the File list

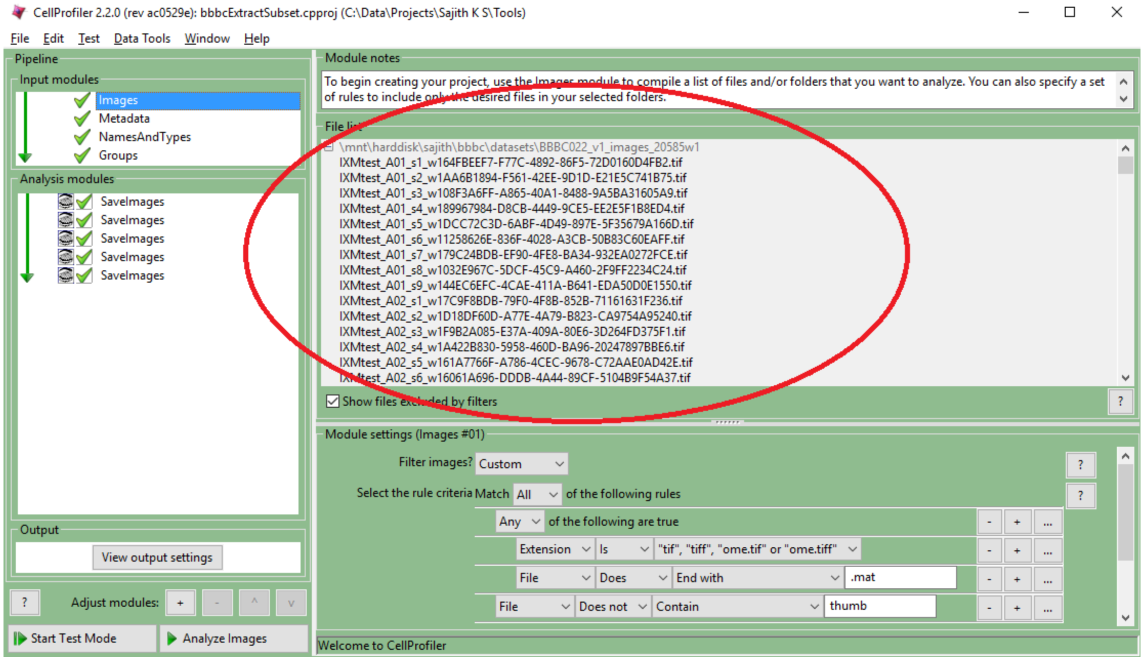

Metadata: Define Metadata file location

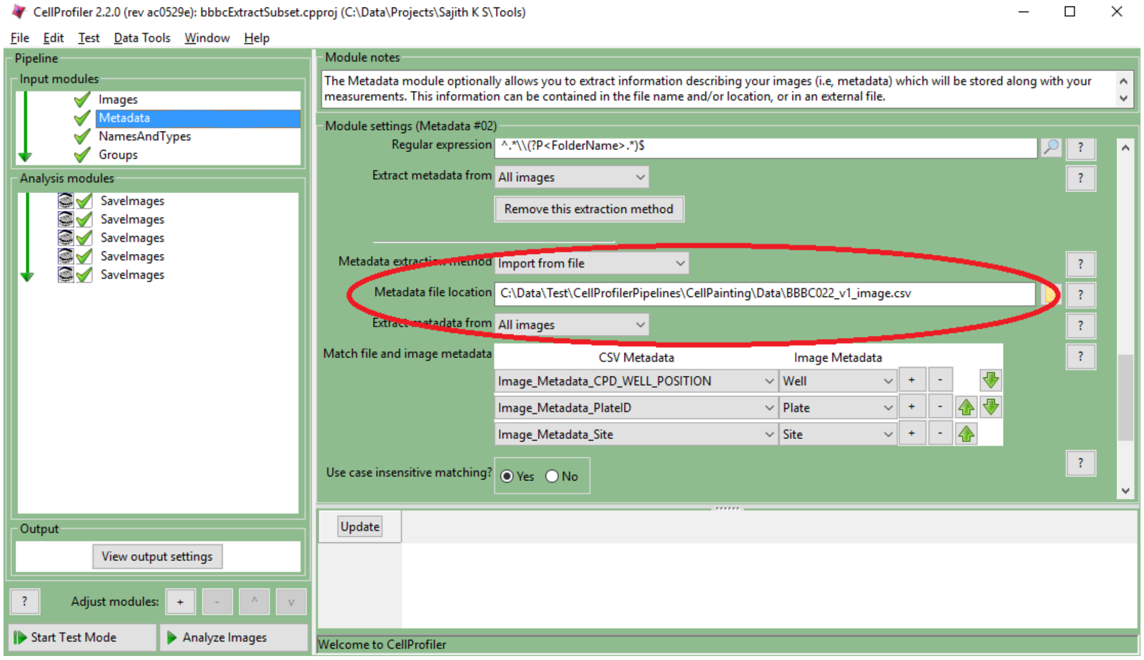

NamesAndTypes: Here we decided only to use the untreated images (mock).

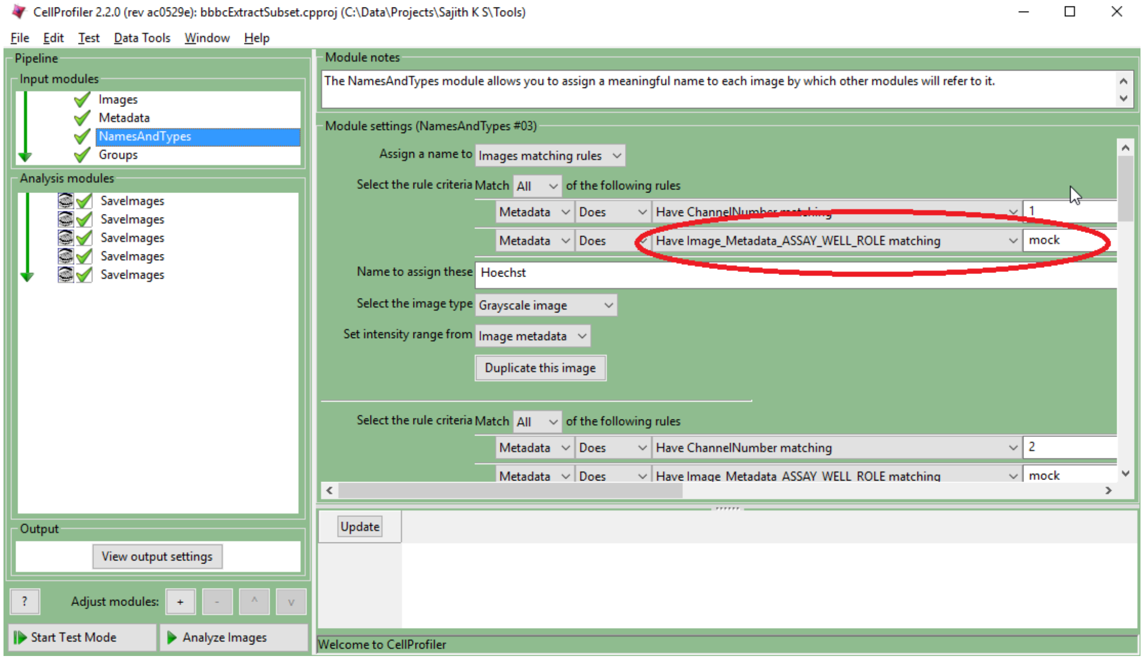

View output settings: Define Default Input Folder to the folder that contains all your image folders. Define Default Output Folder to a folder where your selected images will be copied. The folder structure from the Default Input folder will be created in the Default Output Folder.

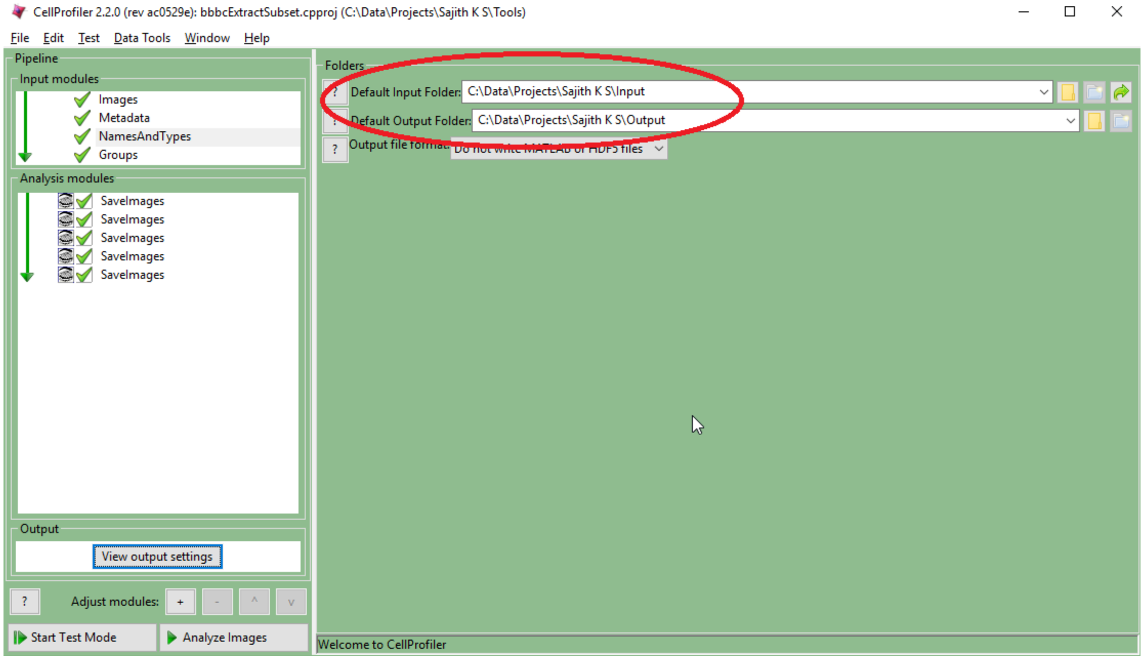

bbbcTrainsetCreatePipeline.cpproj

Images: Drag-and-drop your training images to the File list.

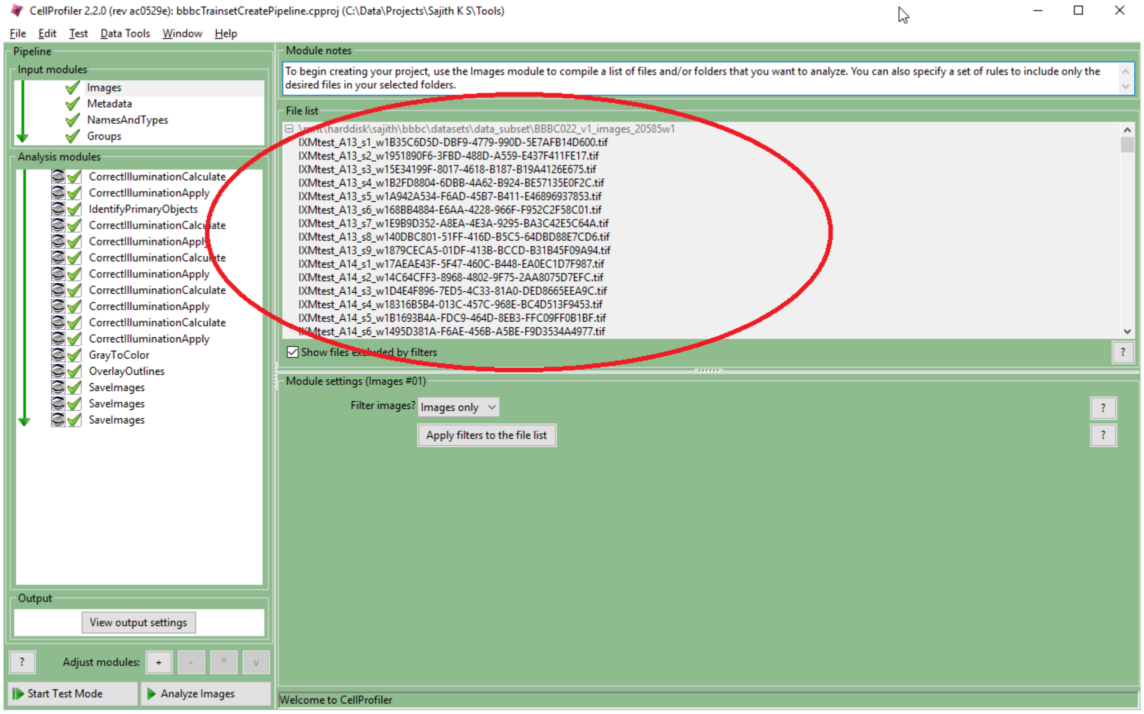

NamesAndTypes: Images from the Evalset should not be included, here Site≠5.

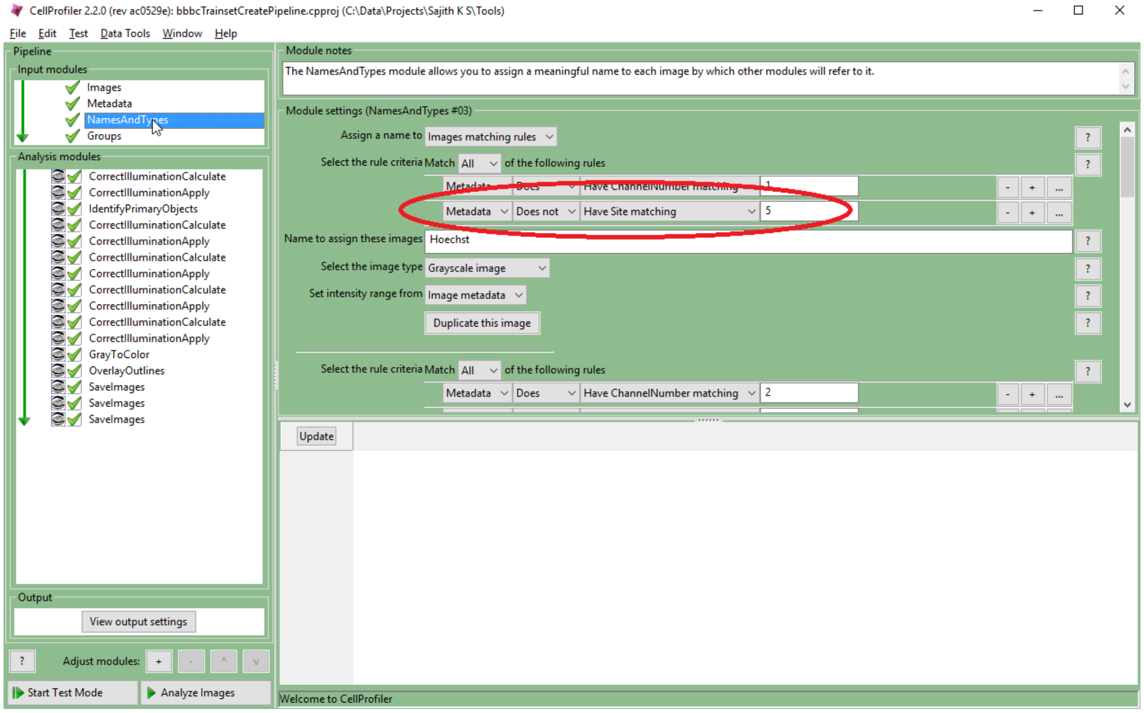

SaveImages #18-20: Define output folders for Stackchannels, Nuclei, OrigOverlay.

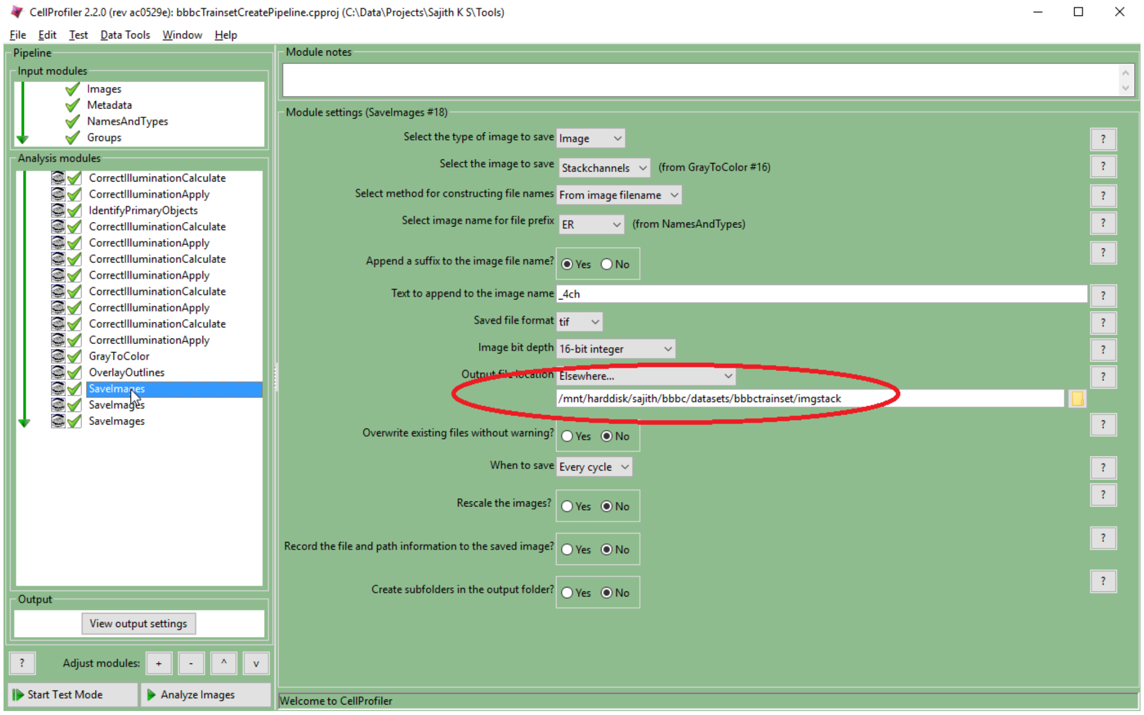

bbbcEvalsetCreatePipeline.cpproj

Images: Drag-and-drop your training images to the File list

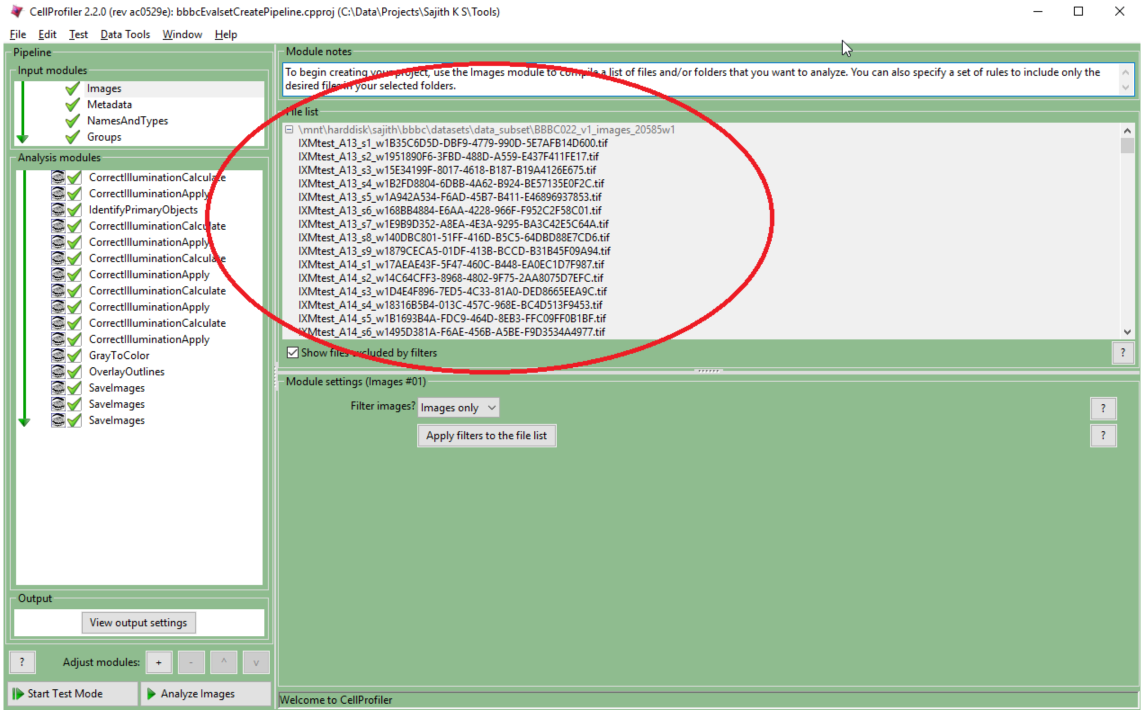

NamesAndTypes: Only images from the Evalset should be included, here Site=5.

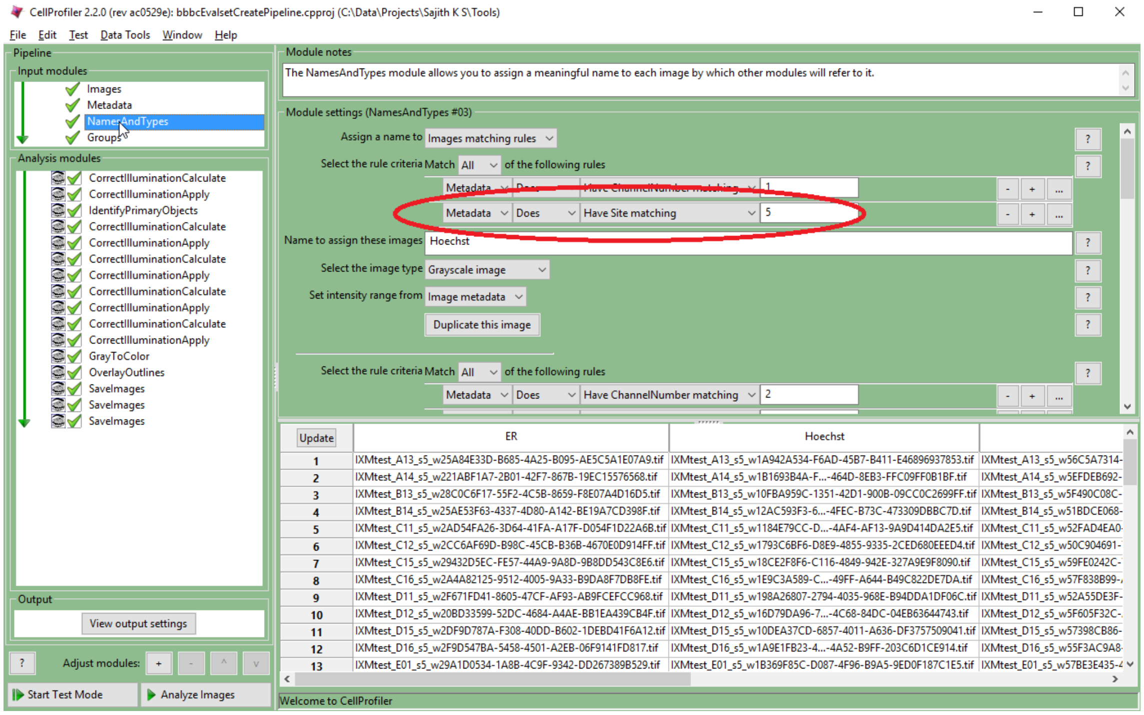

All preprocessing steps (i.e., the steps up to GrayToColor #16) should be the same as in bbbcTrainsetCreatePipeline.

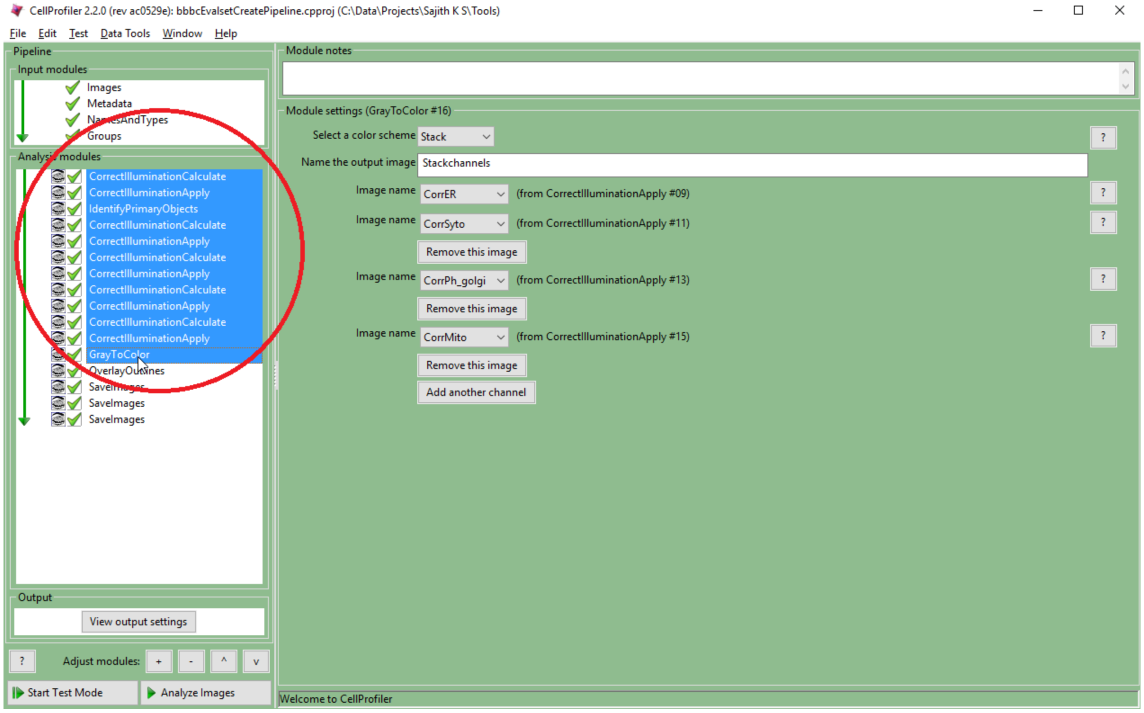

SaveImages #18-20: Define output folders for Stackchannels, Nuclei, OrigOverlay. These should be different folders from the corresponding folders in bbbcTrainsetCreatePipeline.

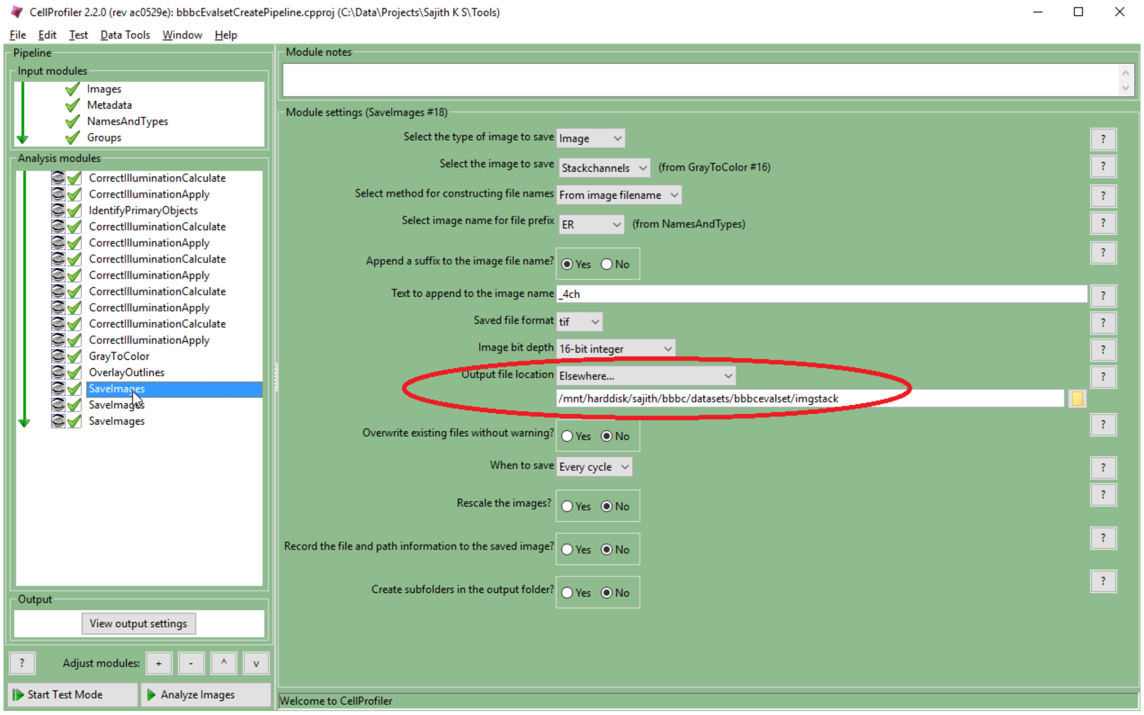

bbbcEvalsetSegmentationPipeline.cpproj

Images: Drag-and-drop your training images to the File list.

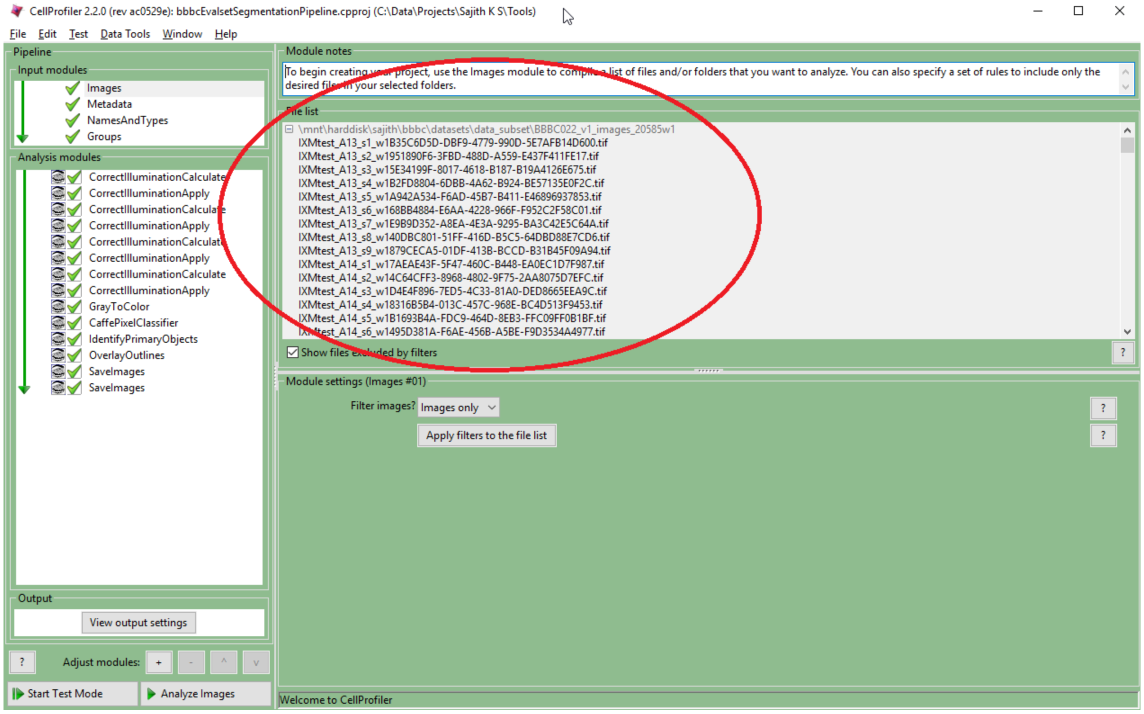

NamesAndTypes: Only images from the Evalset should be included, here Site=5.

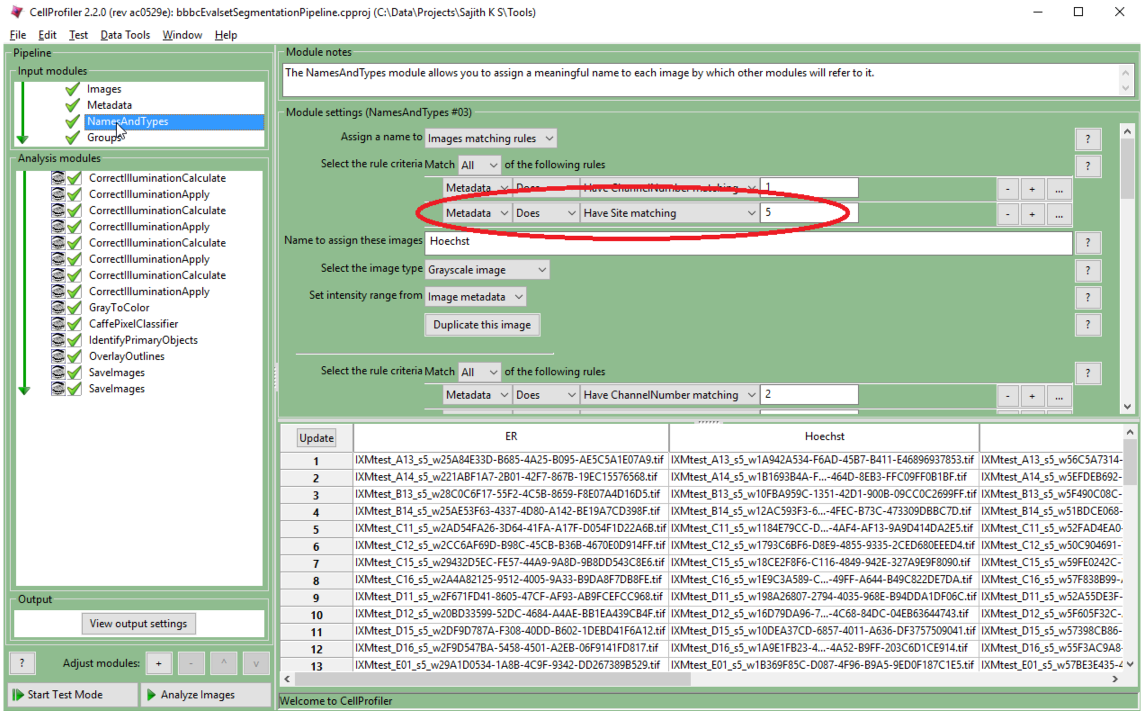

All preprocessing steps (i.e., the steps up to GrayToColor #13) should be the same as in bbbcTrainsetCreatePipeline, except for not usaging the Hoechst channel.

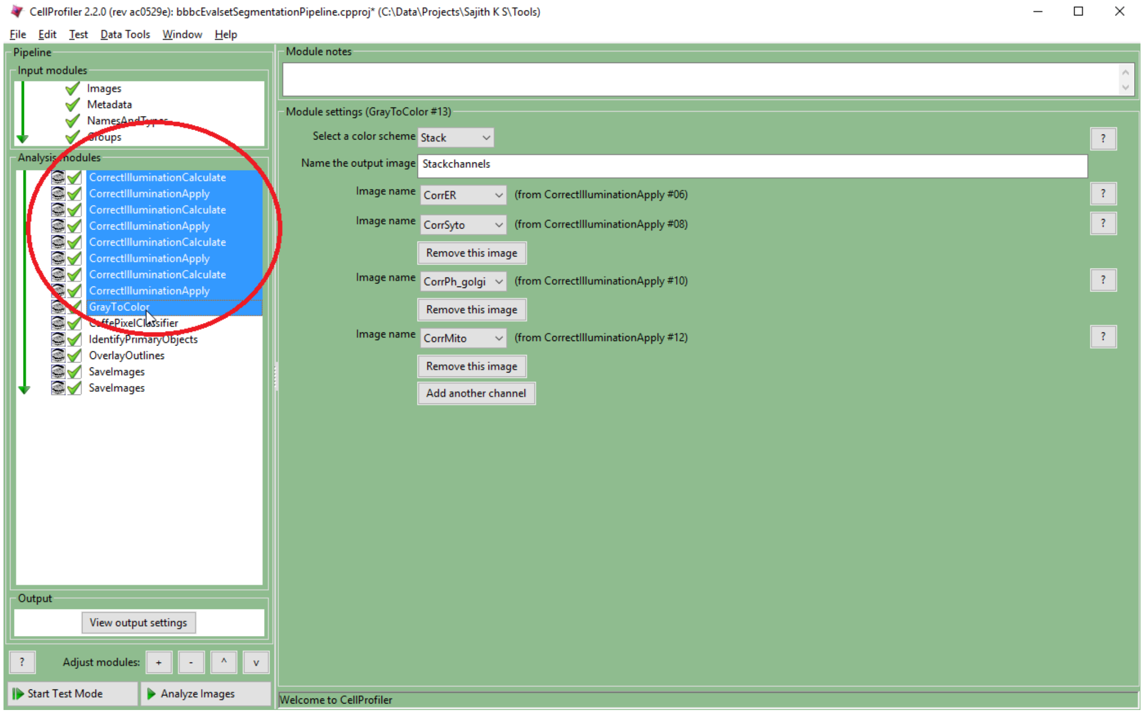

CaffePixelClassifier: Define Caffe root folder location. Define Proto file location. Define Caffe model file location.

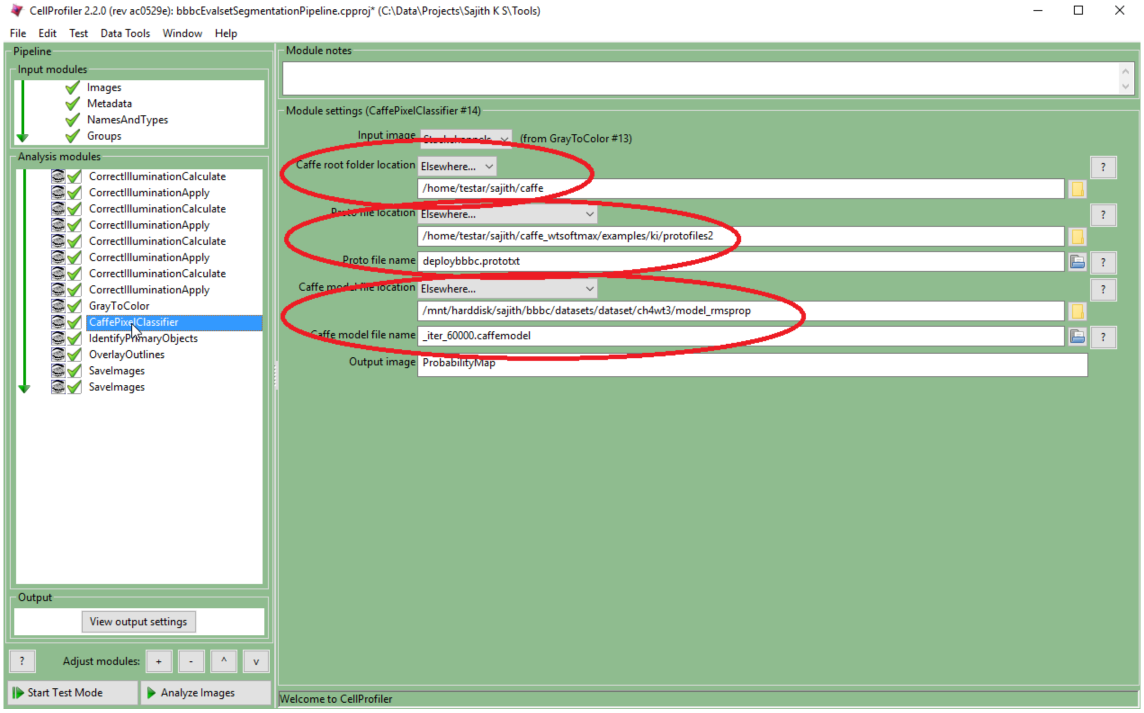

kiTrainsetCreatePipeline.cpproj

Images: Drag-and-drop your training images to the File list.

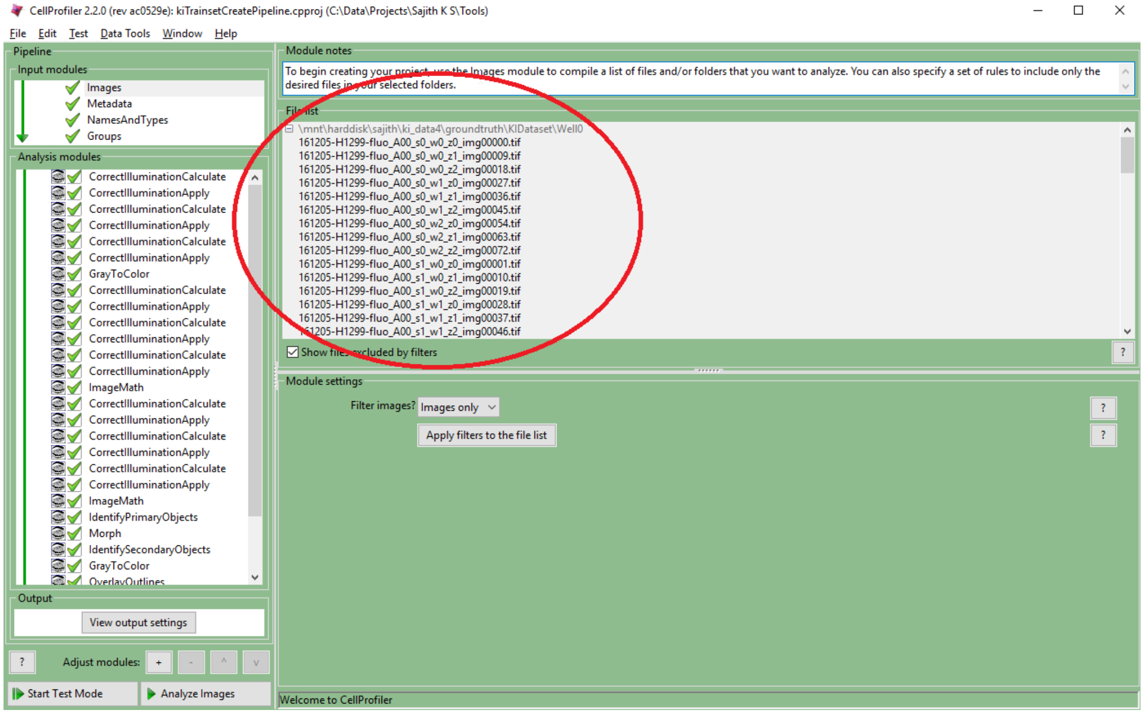

NamesAndTypes: Images from the Evalset should not be included, here Site≠4.

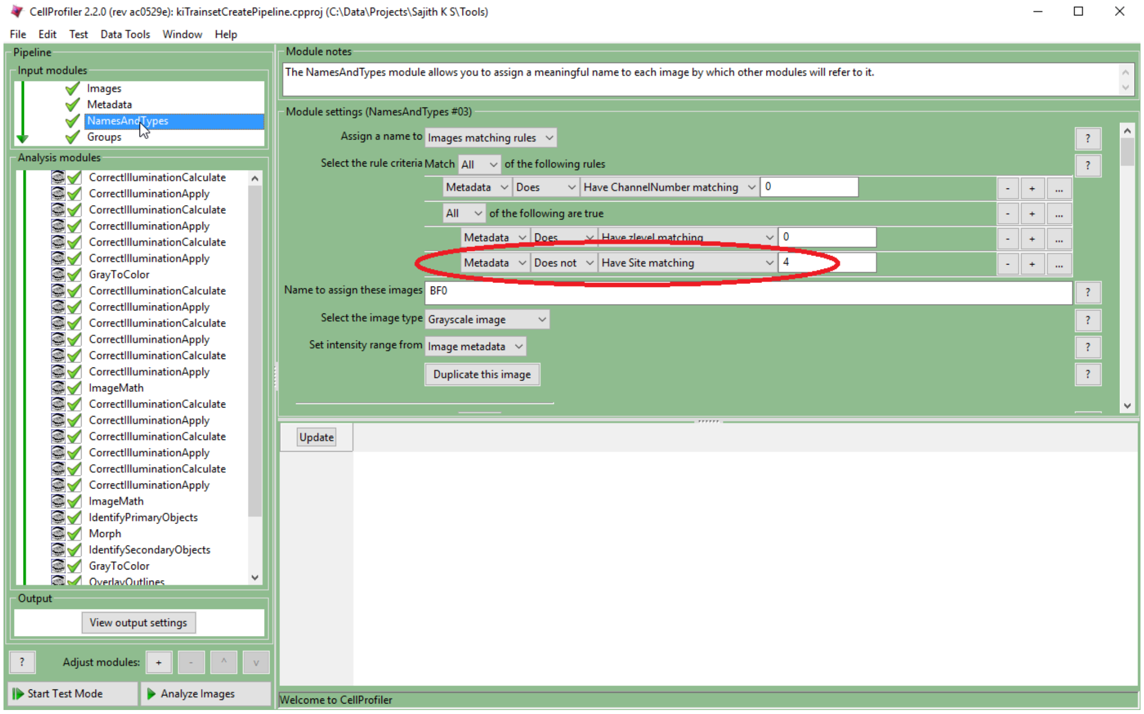

SaveImages #31-33: Define output folders for Cells, BFstack, OrigOverlay.

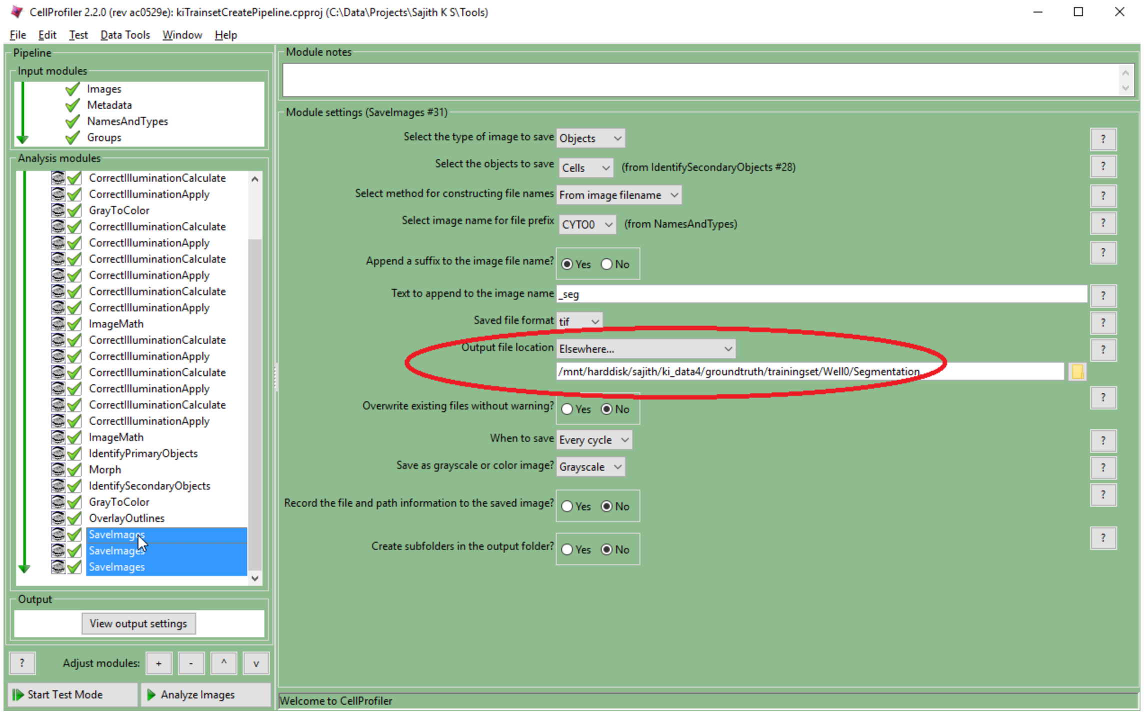

kiEvalsetCreatePipeline.cpproj

Images: Drag-and-drop your evaluation images to the File list.

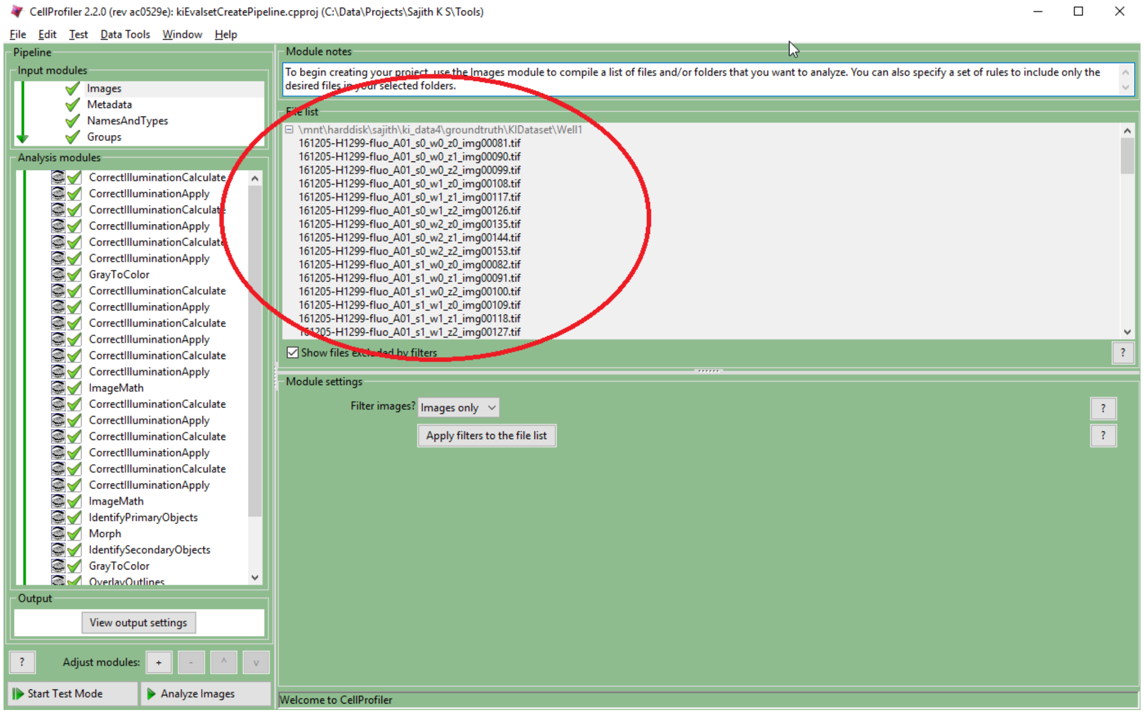

NamesAndTypes: Only images from the Evalset should be included, here Site=4.

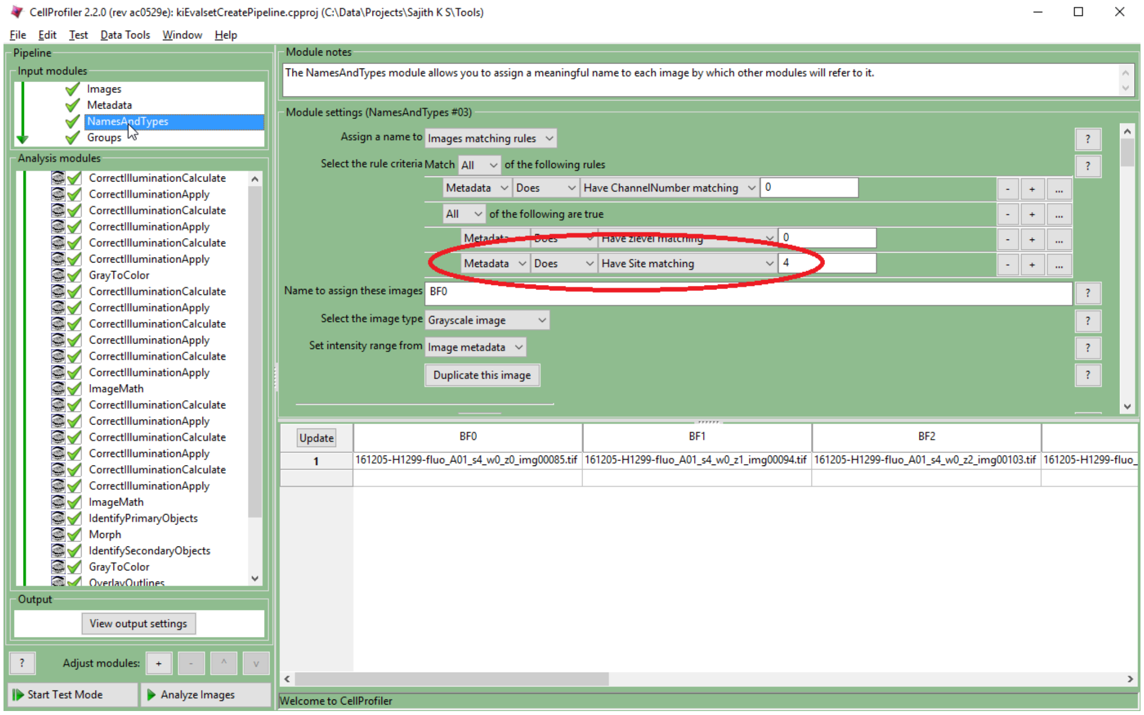

All preprocessing steps should be the same as in kiTrainsetCreatePipeline.

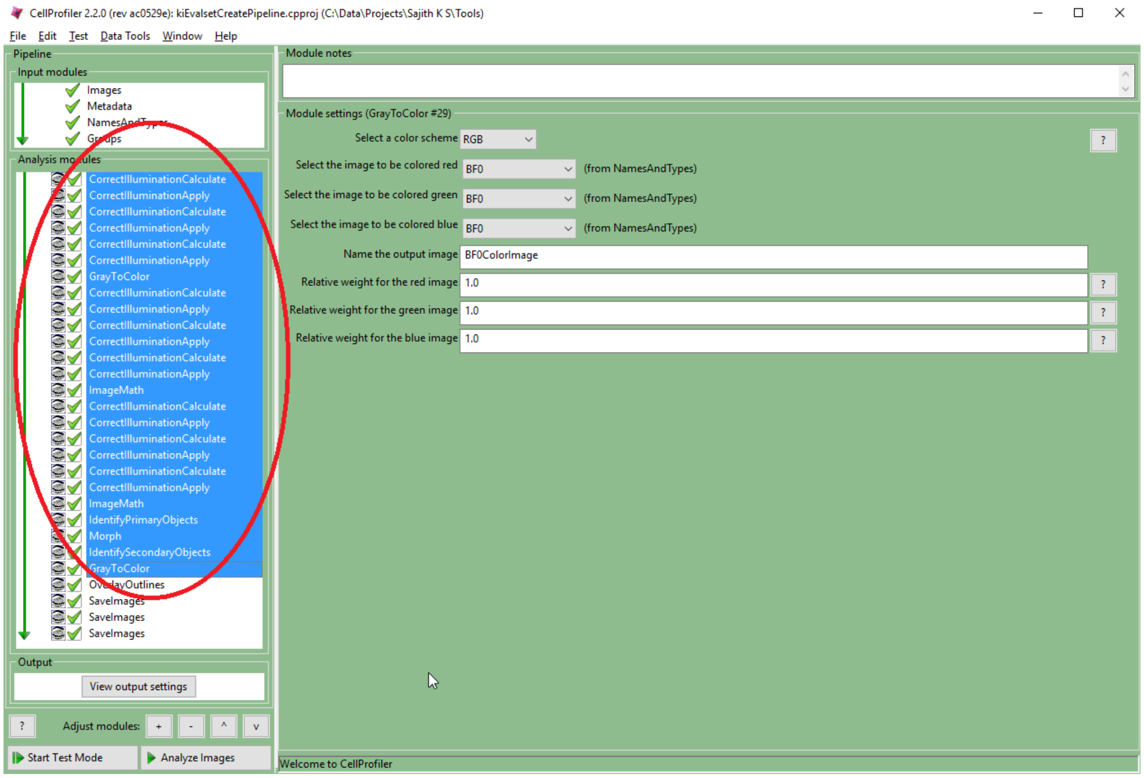

SaveImages #31-33: Define output folders for Cells, BFstack, OrigOverlay. These should be different folders from the corresponding folders in kiTrainsetCreatePipeline.

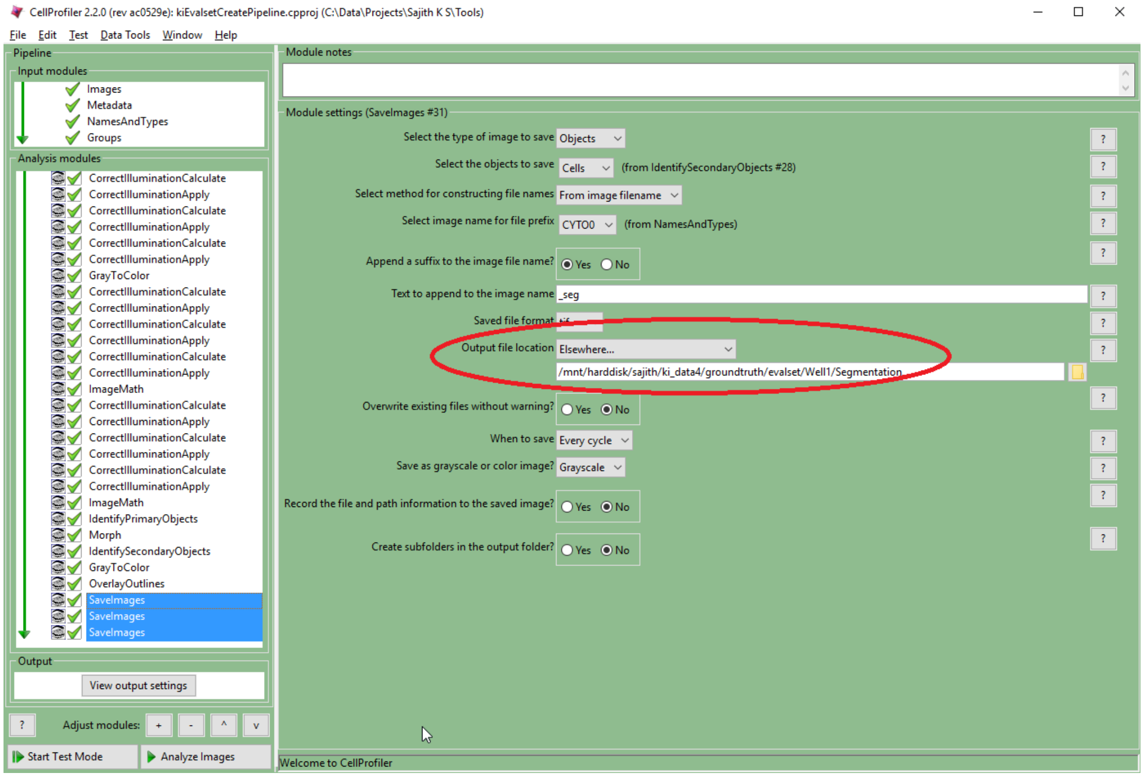

kiEvalsetSegmentationPipeline.cpproj

Images: Drag-and-drop your evaluation images to the File list.

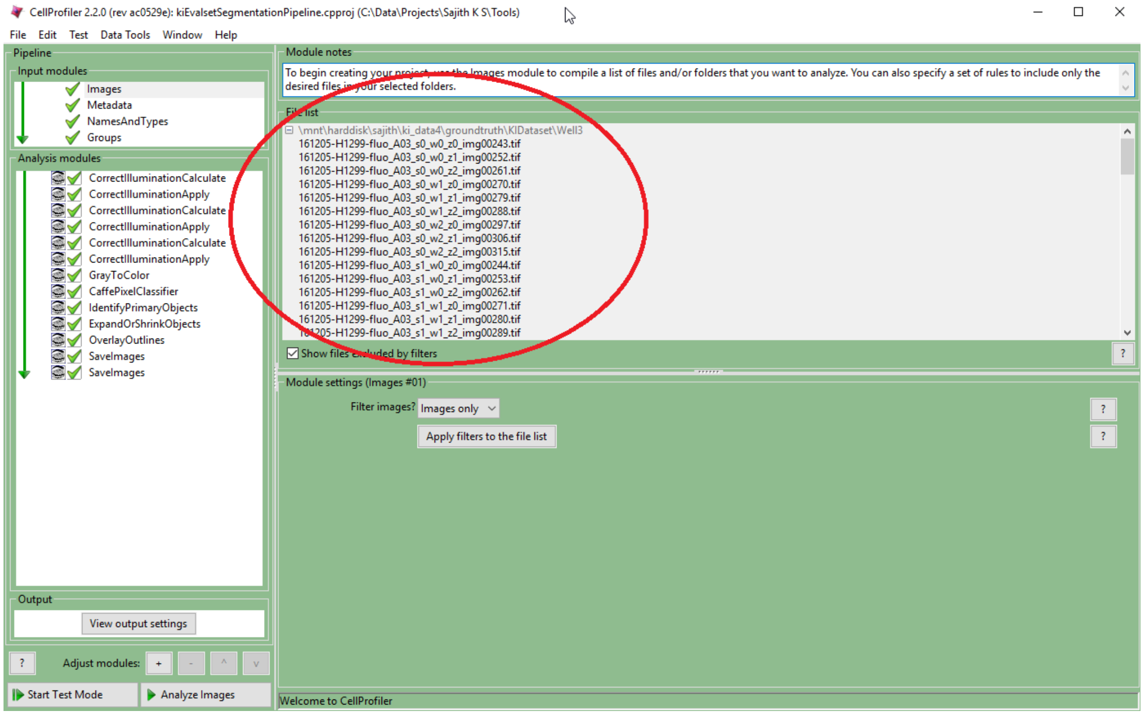

NamesAndTypes: Only images from the Evalset should be included, here Site=4.

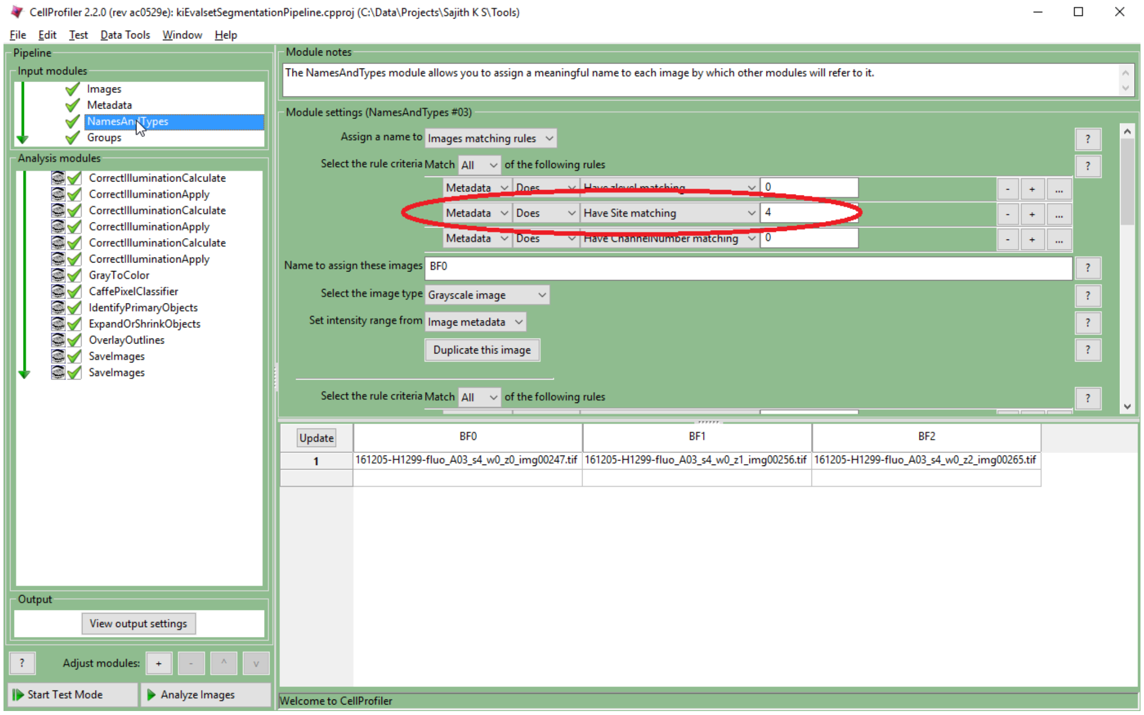

All preprocessing steps (i.e., the steps up to GrayToColor #11) should be the same as in kiTrainsetCreatePipeline.

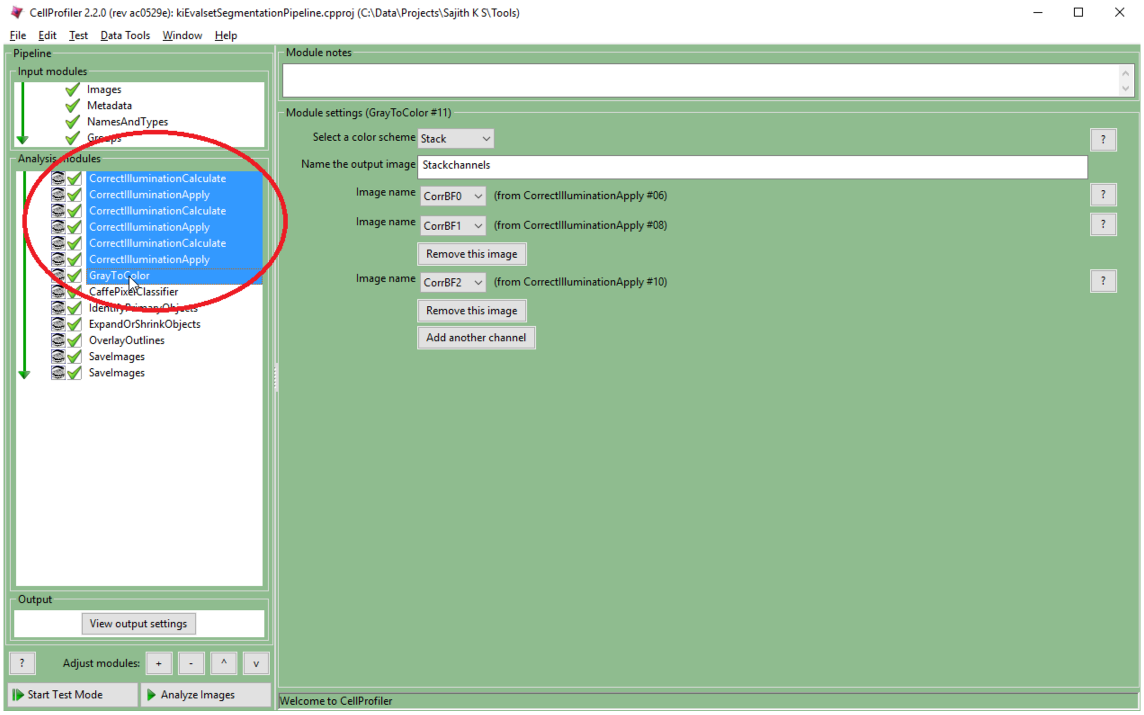

CafePixelClassifier: Define Caffe root folder location. Define Proto file location. Define Caffe model file location.

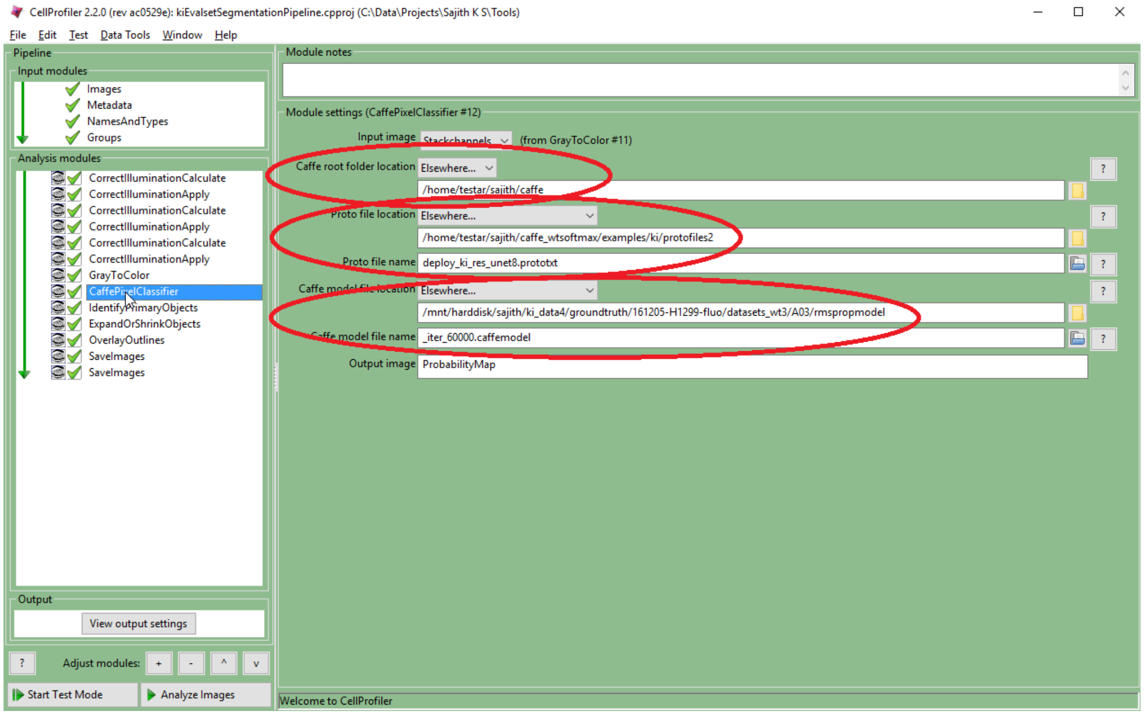

SaveImages #16-17: Define output folders for networkout, OrigOverlay.

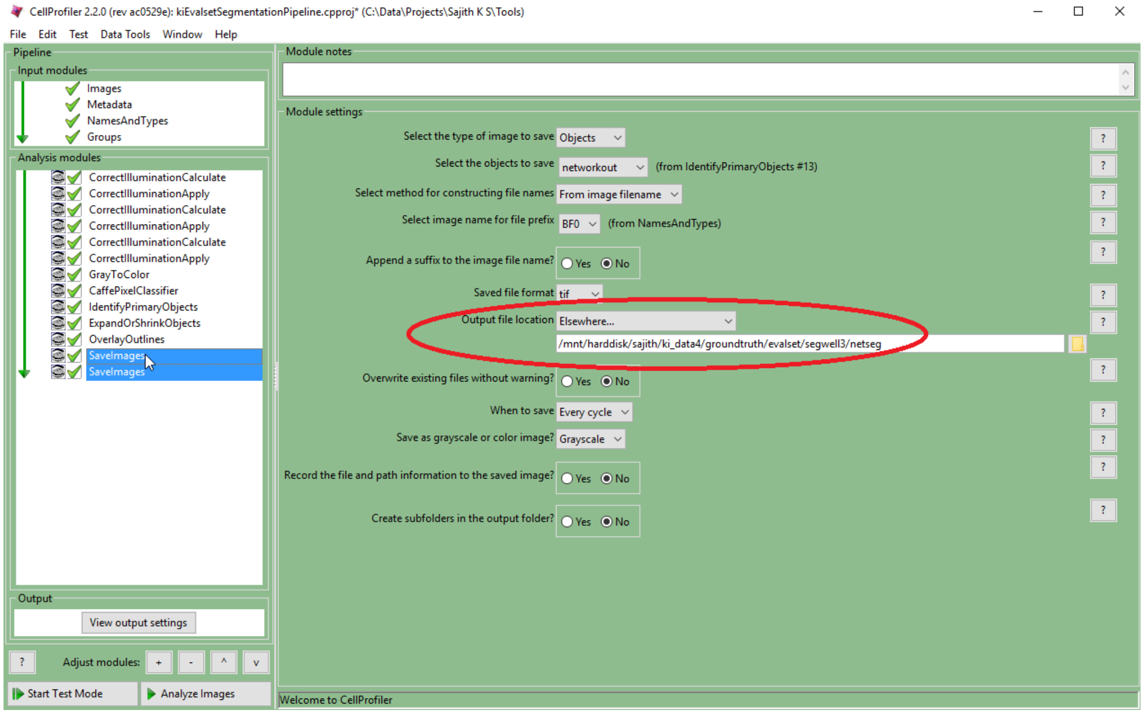

kiSequenceSegmentationPipeline.cpproj

Images: Drag-and-drop your sequence images to the File list.

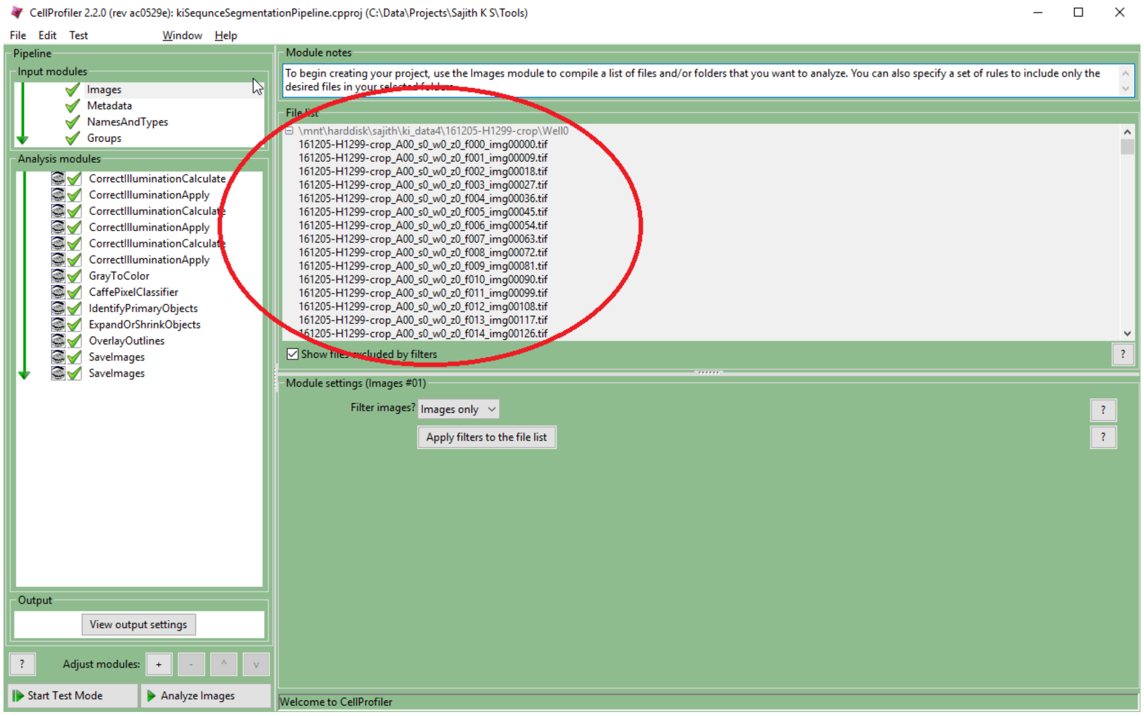

All preprocessing steps (i.e., the steps up to GrayToColor #11) should be the same as in kiTrainsetCreatePipeline.

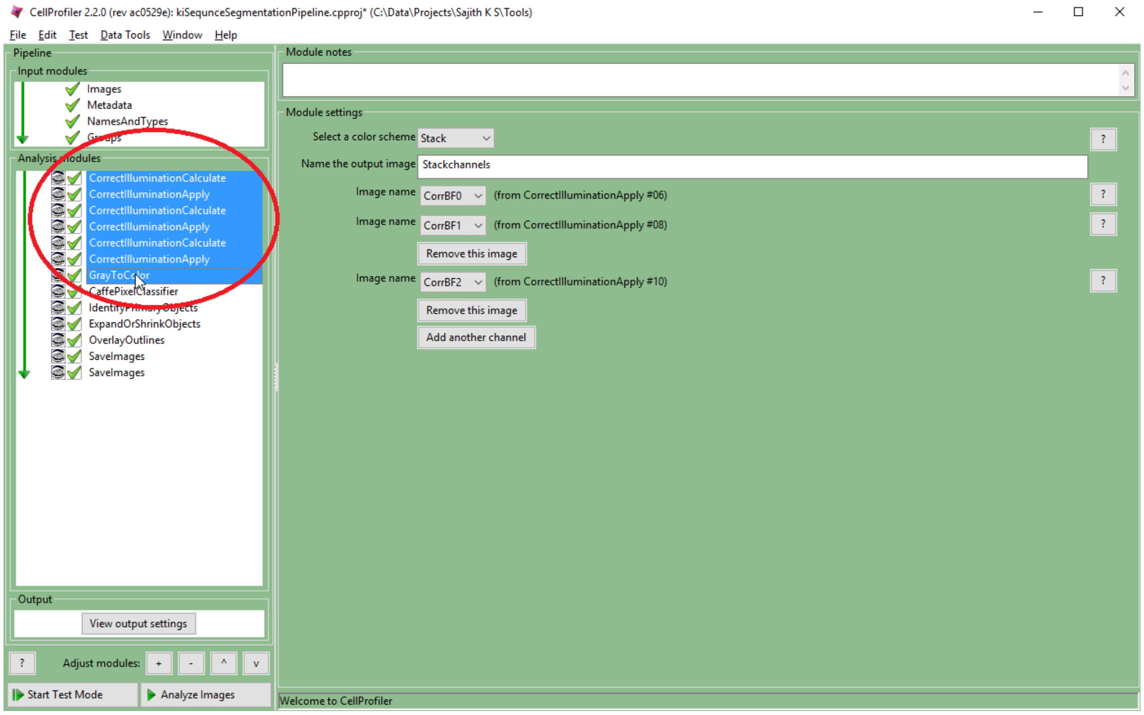

CaffePixelClassifier: Define Caffe root folder location. Define Proto file location. Define Caffe model file location.

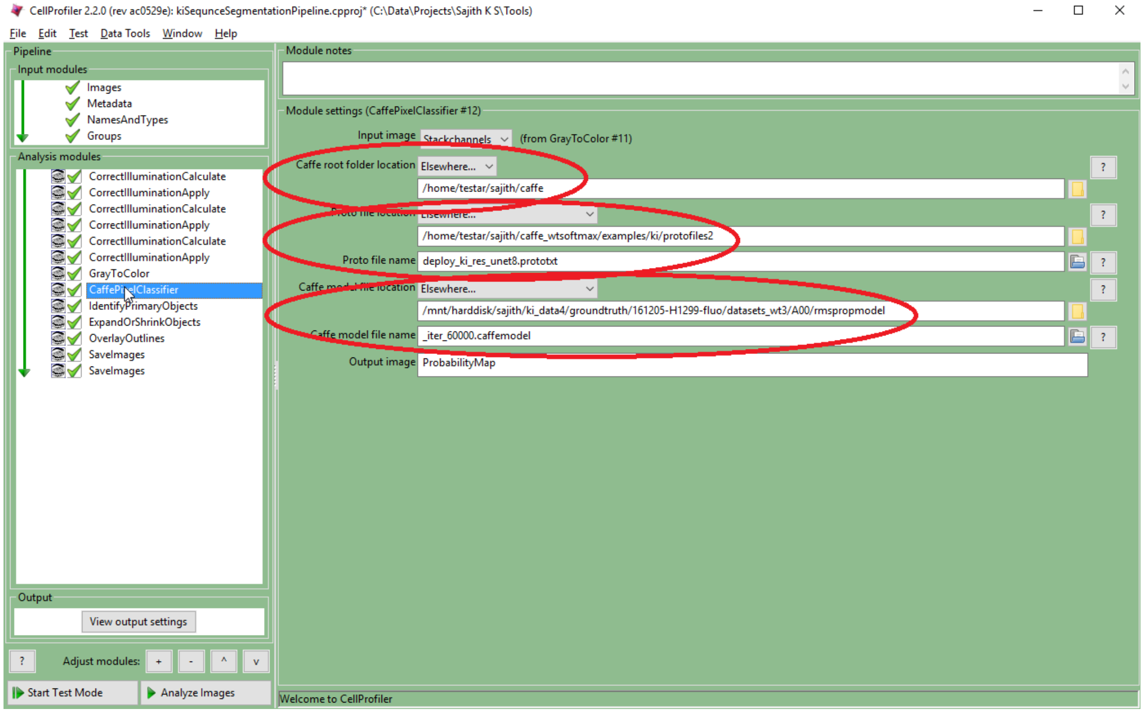

All postprocessing steps (i.e., the steps after to CaffePixelClassifier #12) should be the same as in kiEvalsetSegmentationPipeline.

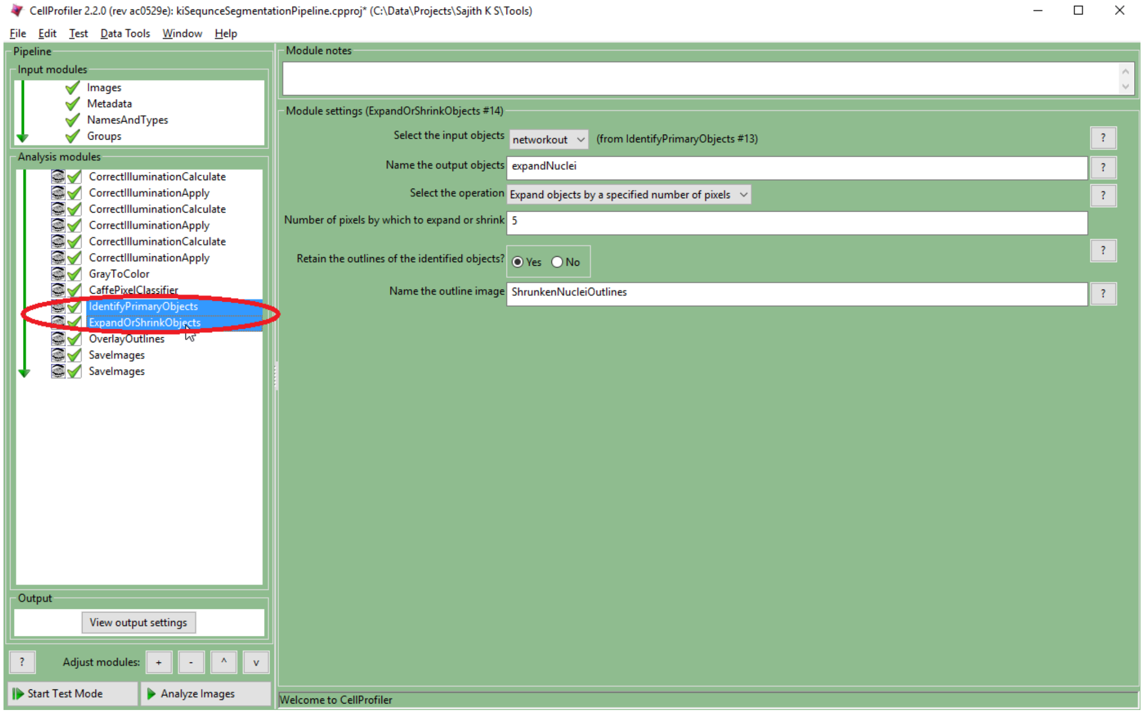

SaveImages #16-17: Define output folders for networkout, OrigOverlay.

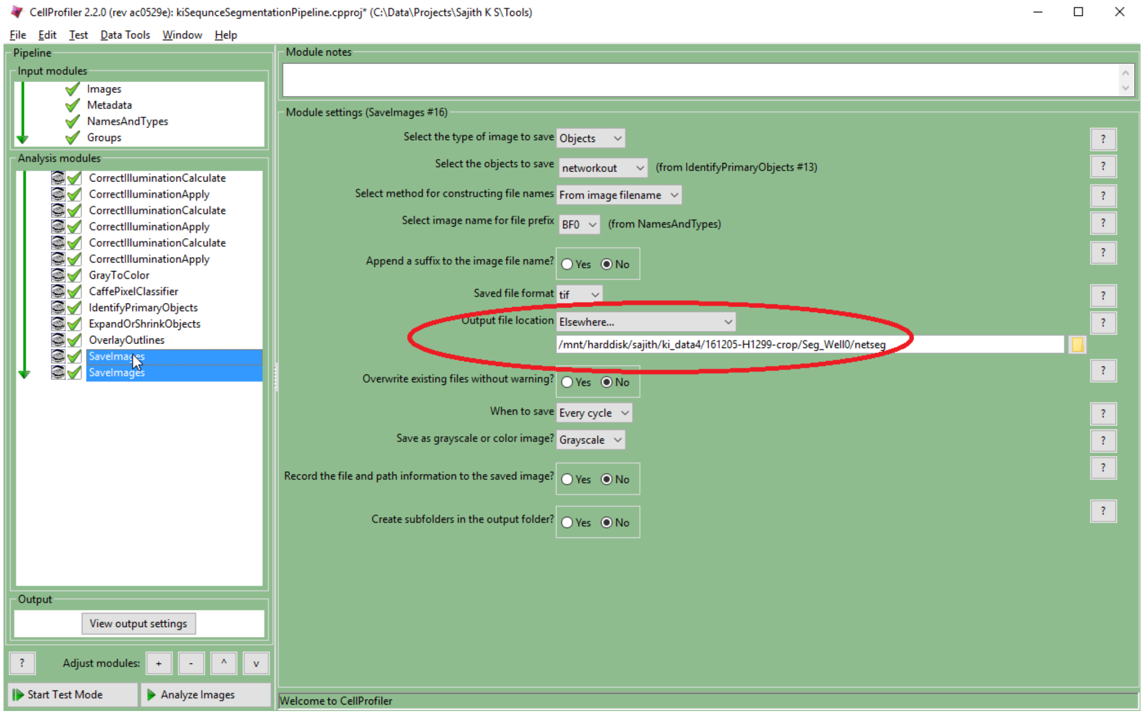

Supplement: Supplementary file 1 — supplementary [file 41598_2017_7599_MOESM1_ESM.pdf]
